# Integration of transcriptome profiling to identify key genes involved in the interplay between oxidative stress and mitophagy in major depressive disorder, followed by multidimensional phenotypic validation

**DOI:** 10.3389/fpsyt.2026.1814473

**Published:** 2026-06-19

**Authors:** Haiyan Fang, Guangjun Zhu, Jin Chen, Tao Zou

**Affiliations:** 1 Clinical Research Center of The Affiliated Hospital of Guizhou Medical University, Department of Clinical Medicine, Guizhou Medical University, Guiyang, China; 2 Department of Psychiatry, The Affiliated Hospital of Guizhou Medical University, Guiyang, China

**Keywords:** CCT3, eEF2, EIF3i, major depressive disorder, mitophagy, oxidative stress

## Abstract

**Background:**

Major depressive disorder (MDD) is recognized as a pressing global public health burden. However, its molecular mechanisms remain incompletely understood.

**Methods:**

In this study, an integrative analysis of transcriptome datasets from the GEO database was conducted. GEO2R and the R programming language were used to identify differentially expressed genes (DEGs) related to oxidative stress and mitophagy. Key hub genes, such as *EEF2*, *CCT3*, *EIF3I*, and *RPS5*, were further identified through enrichment analysis and protein–protein interaction (PPI) network construction. Following validation using an independent human dataset, we established a corticosterone-induced C8-D1A cell model. Reactive oxygen species and mitochondrial membrane potential were measured via flow cytometry. The results demonstrated that this model reliably recapitulates key pathological features of elevated oxidative stress and mitochondrial dysfunction in MDD. Finally, using an *in vivo* mouse model, we assessed synapse-associated proteins and mitophagy markers using Western blotting and measured the mRNA expression levels of candidate genes by qPCR to comprehensively validate the associations between the expression of the aforementioned genes and oxidative stress, mitophagy, and synaptic damage.

**Results:**

This study combined bioinformatics screening and multidimensional phenotypic validation to construct an MDD-specific molecular regulatory network focused on carbon metabolism, thereby elucidating the interplay between four genes and oxidative stress and mitophagy. Although *CCT3* and *RPS5* demonstrated modest diagnostic utility in the independent validation dataset (AUC ≈ 0.6, *Padj* < 0.05), subsequent *in vivo* experiments revealed that the mRNA expression levels of these genes were significantly downregulated in MDD models (*EEF2*: *P* < 0.05; *CCT3*: *P* < 0.005; *EIF3I*: *P* < 0.05). Furthermore, the expression levels of these genes were positively correlated with those of synaptic proteins and negatively correlated with those of mitophagy markers. The downregulation of these genes may impair protein synthesis and folding, which acts in synergy with oxidative stress and mitochondrial dysfunction to perpetuate the vicious cycle of bioenergetic crisis and proteostasis collapse in MDD.

**Conclusion:**

Although this study did not experimentally validate the regulatory functions of the target genes or identify highly specific diagnostic biomarkers, it offers a novel molecular perspective for deciphering the complex pathology of MDD. Notably, this highlights the synergistic interaction between translational regulation and metabolic homeostasis. Further validation in larger independent cohorts is warranted to assess the viability of these genes as mechanistic therapeutic targets.

## 1 Introduction

Major depressive disorder (MDD) is a prevalent global health concern with high recurrence and disability rates. It is clinically characterized by persistently depressed mood, anhedonia, and cognitive impairment, along with diverse somatic symptoms, leading to significant deficits in patients’ social functioning and quality of life (1, 2). Epidemiological data reveal that more than 350 million individuals worldwide suffer from MDD, resulting in a substantial disease burden. In terms of years lived with disability (YLDs), MDD ranks as the second leading cause of global disability, while in terms of disability-adjusted life years (DALYs), it ranks 15th among causes of the global disease burden (3). The dual burden of high prevalence and substantial economic loss stands in stark contrast to the severe imbalance in the global allocation of mental health resources, resulting in significant geographic disparities in the treatment gap. A 2021 study examining service coverage for major depressive disorder (MDD) across 204 countries and regions revealed that only 9.1% of patients globally received minimally adequate treatment, with coverage rates decreasing to as low as 2.0% in low-income regions (4, 5). Current diagnosis relies mainly on the taxonomic criteria of the DSM-5-TR and ICD-11. Major depressive disorder (MDD) has a highly heterogeneous pathological mechanism, and a total of 227 distinct symptom combinations can meet the diagnostic thresholds of the DSM-5-TR, thereby leading to low clinical recognition rates. Recently, both diagnostic systems have increased their emphasis on biological dimensions. The DSM-5-TR incorporates specifiers such as “with mixed features” to refine diagnostic subtypes, whereas the ICD-11 employs a semantic knowledge framework to facilitate the future integration of biomarker data (4, 6). The translation−regulatory genes identified in this study provide molecular evidence for refining such diagnostic systems, thereby aligning with the evolving trends in diagnostic criteria.

Abnormal activity of the hypothalamic–pituitary–adrenal (HPA) axis is among the core biological characteristics of major depressive disorder (MDD) (1). HPA axis hyperactivity has been established as a crucial pathophysiological mechanism underlying MDD (7). Juruena (8) reported that stressful life events, such as childhood emotional abuse, physical abuse, and sexual abuse, can trigger hyperactivity of the HPA axis and an increase in circulating cortisol levels. Hypercortisolemia plays a central role in stress−related neuropathologies, including depression (9). Astrocytes play pivotal roles in the mechanisms underlying stress-related neuropathology, depressive susceptibility, and disease persistence. Specifically, astrocytic dysfunction acts as a crucial mediator between chronic stress and impaired neural plasticity (10, 11). Growing evidence from studies on major depressive disorder (MDD) indicates that disruption of the astrocyte–neuron glutamate–glutamine cycle, particularly a homeostatic imbalance within the medial prefrontal cortex, may precipitate the accumulation of reactive oxygen and nitrogen species and ultimately induce depressive-like behaviors (12). Furthermore, large-scale clinical studies have confirmed that patients with MDD generally exhibit elevated oxidative stress levels, manifested by increased lipid peroxidation and oxidative protein damage, as well as significantly reduced levels of antioxidant enzymes and nonenzymatic antioxidants (13–15). An imbalance in the oxidative−antioxidant system is among the key pathological hallmarks of major depressive disorder (MDD) (16, 17). Furthermore, the *de novo* synthesis of glutamine in astrocytes during the glutamate–glutamine cycle consumes a substantial amount of energy through the generation of α−ketoglutarate from the TCA cycle (18). Glutamate uptake is also an energy-intensive process (19). Consequently, mitochondrial dysfunction severely compromises astrocytes within the glutamate-glutamine (Glu-Gln) cycle. Mitochondrial quality control forms the fundamental regulatory network for maintaining mitochondrial function. Among these processes, mitophagy serves as a pivotal mechanism for eliminating damaged mitochondria under stress and sustaining the cellular energy supply. Mitophagic dysregulation plays a pivotal role in the pathogenesis of depression. Recent studies have demonstrated that drugs targeting mitophagy hold considerable potential for treating neurodegenerative diseases and aging, thereby positioning them as promising therapeutic targets with substantial value for research and clinical translation (20). The specific types of crosstalk among major depressive disorder (MDD), oxidative stress and mitophagy require further elucidation.

By mining public databases and performing bioinformatic analyses, this study identified differentially expressed genes (DEGs) between patients with MDD and healthy controls. Venn diagram analysis was employed to identify overlapping DEGs associated with oxidative stress and mitophagy. Subsequent analyses included functional enrichment analysis and the identification of hub genes within the protein–protein interaction (PPI) network, which was subsequently visualized using Cytoscape software (version 3.10.3) (21). The candidate genes were identified via the CytoHubba plugin (22). These genes may contribute to the pathophysiological mechanisms of MDD and act as potential biomarkers linked to oxidative stress and mitophagy. Candidate genes were validated using an independent human validation cohort and a corticosterone-induced depressive mouse model. This study aims to identify key predictive factors for the development and progression of MDD and to explore novel multitarget synergistic intervention strategies informed by these findings.

## 2 Materials and methods

### 2.1 GEO dataset

The dataset was obtained from the NCBI GEO database (https://www.ncbi.nlm.nih.gov/geo/) with the search terms “major depressive disorder” and “RNA expression”. The GSE52790 dataset, deposited by Li et al., was downloaded from the GEO database and served as the training set. This dataset was generated using the Affymetrix Human hGlue_3_0_v1 Array platform and included 10 patients with major depressive disorder (MDD) and 12 age- and sex-matched healthy controls. The original metadata of this dataset did not provide data on the medication history of the participants. A total of 22 samples were included in this dataset and acquired from public databases. The GSE98793 dataset, generated using the Affymetrix Human Genome U133 Plus 2.0 Array platform, was used as the validation set and comprises 168 patients with major depressive disorder (MDD) and 64-well-matched healthy controls. Given the retrospective nature of the study, informed consent and ethics committee approval were not needed.

### 2.2 DEG identification

Differentially expressed genes (DEGs) in the training dataset GSE52790 were identified using the online tool GEO2R (23). Genes whose ∣Log2FC∣>1 and *P* value<0.05 were classified as differentially expressed for subsequent analysis. Gene annotation files were downloaded from the Ensembl database (24) (https://www.ensembl.org/), and four lncRNAs were excluded. Analyses were performed using R version 4.2.1, and the R package ggplot2 (version 3.4.4) was employed for subsequent analyses and the visualization of PCA plots. In addition, phenotype-related gene sets related to mitophagy and oxidative stress were retrieved on September 8, 2025, from GeneCards: The Human Gene Database (https://www.genecards.org/) (25) using the keywords “mitophagy” and “oxidative stress”. The relevance score was thresholded at ≥ 1, and the category was restricted to “protein coding”. After these criteria were applied, a total of 1716 mitophagy−related genes and 3056 oxidative stress−related genes were retained. These gene lists were cross-referenced with the differentially expressed genes (DEGs) from the GSE52790 dataset to identify DEGs linked to mitophagy and oxidative stress. The R packages ggplot2 (v3.4.4), VennDiagram (v1.7.3), and ComplexHeatmap (v2.13.1) were employed to generate Venn diagrams and heatmaps, respectively.

### 2.3 Functional enrichment analysis

Functional enrichment analyses of the DEGs were conducted via the Xiantao Academic Online Platform (https://www.xiantaozi.com). Venn diagrams were generated from the DEGs associated with mitophagy, oxidative stress, and MDD to identify common coexpressed genes. Subsequent GO enrichment analysis (covering biological processes [BP], cellular components [CC], and molecular functions [MF]) and Kyoto Encyclopedia of Genes and Genomes (KEGG) pathway analysis, along with their visualization, were performed using the following parameters: Min Overlap = 3, *P* value cutoff = 0.05, and Min Enrichment = 1.5.

### 2.4 Protein–protein interaction network analysis

The list of differentially expressed genes linked to mitophagy and oxidative stress was submitted to the STRING database for protein–protein interaction analysis (https://string-db.org) (26). Construction of the protein–protein interaction (PPI) network: The search was restricted to “*Homo sapiens*” within the STRING database interface, and the minimum interaction confidence score was set to the medium threshold (≥ 0.400). This threshold filters out low-confidence predicted interactions, retaining only those pairs with moderate or higher confidence derived from multiple sources, such as experimental validation, database predictions, and literature mining. This approach ensures sufficient network coverage while minimizing background noise. Following data retrieval, the interaction tables and network files (in TSV format) were exported and subsequently imported into Cytoscape (version 3.10.3) for PPI network visualization and topological analysis. The MCC algorithm, available within the CytoHubba plugin, was employed to identify the top 10 hub genes on the basis of their degree scores; these genes were then selected as candidate targets for subsequent functional enrichment and mechanistic validation (27).

### 2.5 Candidate gene diagnosis model construction

In this study, preliminary screening and diagnostic efficacy evaluation of candidate genes were conducted via the diagnostic ROC module of the online XianTaoZi platform (https://www.xiantaozi.com). After confirming consistent expression trends, an adjusted *P* value (*P.adj*) < 0.05 was set as the threshold for statistical significance. Additional validation was performed using the independent external dataset GSE98793 (containing peripheral whole-blood transcriptome data from 128 patients with major depressive disorder (MDD) and 64 healthy controls). Batch effect correction was performed using the ComBat algorithm from the R “sva” package. Multiple testing correction was performed using the Benjamini–Hochberg (BH) method with m=4, which was applied exclusively to the testing of candidate genes. Then, the pROC package was employed to generate receiver operating characteristic (ROC) curves for each candidate gene exhibiting significant differential expression in the validation set to calculate the area under the curve (AUC) and its 95% confidence interval (CI) to evaluate the diagnostic performance and generalizability of the findings. (The detailed code is provided in the **Supplementary Material**).

### 2.6 Experimental validation

#### 2.6.1 Cell experiments

##### 2.6.1.1 Grouping and viability of the C8-D1A cell line

Astrocytes function as pivotal metabolic regulators in the brain. Their crucial roles in maintaining the physiological homeostasis of the nervous system are mediated primarily by the regulation of glutamate excitotoxicity, antioxidant defense, and cerebral energy metabolism (28–30). The C8-D1A cell line (Pricella, China) is a spontaneously immortalized cell line derived from mouse cerebellar astrocytes. After cell resuspension, the cells were cultured under standard conditions. Cells were maintained in high-glucose DMEM supplemented with 10% fetal bovine serum (FBS) and 1% penicillin-streptomycin (P/S) and incubated at 37 °C in a humidified incubator with 5% CO_2_ and 95% ambient air. Cells in the logarithmic growth phase were used for all experiments. Cells were seeded into each well of a 96−well plate (10,000 cells/well) and incubated at 37 °C. After 24 h, 200 μL of the corticosterone solution at concentrations ranging from 0 to 800 μmol/L (0, 50, 100, 200, 400, 800 μmol/L) was added to each well, and the cells were incubated for an additional 48 h. Cell viability was evaluated via the CCK−8 assay. Briefly, 10 μL of CCK−8 solution was added to each well. After incubation at 37 °C for 40 min, the absorbance was measured at 450 nm with a microplate reader (BioTek Instruments, Inc., USA). Cell viability was calculated using the following formula: cell viability (%) = (experimental − blank)/(control − blank) × 100%. Each group consisted of five replicate wells, and all experiments were performed in triplicate.

##### 2.6.1.2 Determining intracellular reactive oxygen species (ROS) levels and mitochondrial membrane potential

Mitochondria are known as the “powerhouses” of cells, and a vicious cycle of bidirectional regulation occurs between mitochondrial damage and reactive oxygen species (ROS) production. Dysfunction of the mitochondrial electron transport chain, particularly Complexes I and III, induces electron leakage, leading to the excessive generation of ROS such as superoxide anions (O_2_^-^) and hydrogen peroxide (H_2_O_2_). Excess ROS further damage mitochondrial inner membrane lipids, respiratory chain proteins, and mitochondrial DNA (mtDNA), resulting in the disruption of membrane integrity, a decrease in mitochondrial membrane potential, impaired ATP synthesis, and exacerbated mitochondrial dysfunction. Therefore, ROS levels serve as a core quantitative indicator of mitochondrial damage (31). Intracellular ROS levels were measured using a DCFH−DA detection kit (Beyotime, China). C8-D1A cells in the logarithmic growth phase were collected and resuspended in DMEM at a concentration of 2.5×10^5^ cells/mL. The cell suspension was seeded into a 6-well plate at 2 mL per well and incubated overnight at 37 °C under 5% CO_2_ in a humidified atmosphere. The cells were assigned to the normal group or the corticosterone−treated model group. The cells were rinsed twice with PBS and then centrifuged at 1500 rpm for 5 min, after which the supernatant was discarded. Subsequently, 1 mL of DCFH solution diluted with serum-free medium at a ratio of 1:1000 was added, and the cells were incubated at 37 °C for 20 min. The suspension was gently agitated every 3 min during incubation. Following three washes with serum-free medium, the cells were resuspended in 500 μL of PBS. The intracellular ROS level was ultimately determined by flow cytometry (Beckman Coulter, USA).

Changes in mitochondrial membrane potential were assessed using a JC-1 detection kit (Beyotime, China). Healthy C8-D1A cells in the logarithmic growth phase were collected and prepared as single-cell suspensions in DMEM, with the cell concentration adjusted to 2.5 × 10^5^ cells/mL. Cells were seeded uniformly into a 6-well plate at 2 mL per well and incubated overnight at 37 °C in a humidified atmosphere containing 5% CO_2_. The cells were divided into a normal group and a corticosterone-treated model group. Subsequently, 0.5 mL of JC-1 staining working solution was added, and the mixture was gently inverted several times to ensure homogeneous mixing. The cells were incubated at 37 °C for 20 min. Concurrently, 1× JC-1 staining buffer was prepared by diluting the 5× stock with distilled water at a 1:4 (v/v) ratio and keeping it on ice. After incubation at 37 °C, the cells were centrifuged at 1200 rpm for 3 min at 4 °C, and the supernatant was discarded. The cells were subsequently washed twice with 1× JC-1 staining buffer. For each wash, the cells were resuspended in 1 mL of buffer and centrifuged at 1200 rpm for 3 min at 4 °C, after which the supernatant was removed. This resuspension and centrifugation step was repeated once 1 mL of fresh 1× JC-1 staining buffer was used under identical conditions, after which the supernatant was removed. Following resuspension in 500 μL of 1× JC-1 staining buffer, changes in JC-1 fluorescence were measured using a flow cytometer (Beckman Coulter, Brea, CA, USA).

#### 2.6.2 Animal experiments

##### 2.6.2.1 Experimental group allocation

This study was conducted in accordance with the internationally accepted ARRIVE 2.0 guidelines regarding experimental design and reporting, thereby fulfilling all 10 essential items. A total of 20 4-week-old C57BL/6 mice (weighing 12 ± 1 g) consisting of an equal number of males and females were purchased from Henan Sikebeisi Biotechnology Co., Ltd. (License No.: SCXK(Yu) 2025-0005). The animal facility environment was maintained at 24 ± 2 °C with a relative humidity of 55 ± 5%. The animals were kept on a 12-hour light/dark cycle. All the mice were provided ad libitum access to purified water and standard laboratory chow. The sample size was calculated *a priori* using GPower 3.1 (Heinrich Heine University Düsseldorf, Germany). For the independent two−sample t−test, the two−tailed significance level (α) was set at 0.05, and the target statistical power was set to 0.8. On the basis of data from preliminary sucrose preference tests, the mean sucrose preference index was 94.5% in the normal control group and 69.7% in the depression model group, yielding a pooled standard deviation (σ) of 0.17. A Cohen’s d effect size of 1.46 was calculated, indicating a required sample size of 6 animals per group. To account for an anticipated 15% rate of modeling failure and animal attrition, an initial sample size of 10 mice per group was determined. The mice were randomly assigned to either a normal control group or a depression model group induced via subcutaneous corticosterone injection. Following a 7-day acclimatization period, control mice were group-housed, whereas model mice were individually housed under controlled experimental conditions. The Experimental Animal Ethics Committee of Guizhou Medical University approved this study (No. NO2502768).

##### 2.6.2.2 Establishment of a model of depression-like behavior

Corticosterone solution was prepared with 0.1% DMSO, 0.3% Tween−80 and normal saline. Briefly, corticosterone was dissolved in a minimal volume of DMSO to prepare a stock solution to a final concentration of 25 mg/mL. The working solution was then diluted to 2.5 mg/mL with a mixed vehicle consisting of 40% PEG300, 5% Tween−80 and 45% normal saline. Mice were subcutaneously injected with exogenous corticosterone at a daily dosage of 20 mg/kg body weight for 3 consecutive weeks to establish a chronic stress state and induce depression−like behaviors, thereby mimicking hypothalamic–pituitary–adrenal (HPA) axis dysfunction caused by chronic stress in humans (1, 32, 33). Body weight was measured every 3 days, and dorsal injection sites were examined daily; animals exhibiting redness, swelling, or induration with a diameter exceeding 3 mm were excluded. The mice in the healthy control group were subcutaneously injected with an equal volume of vehicle containing 0.1% DMSO, 0.3% Tween−80 and normal saline. Behavioral tests were performed before and after the experimental intervention. All the mice were euthanized under deep anesthesia. Anesthesia was induced via inhalation of 3%–5% isoflurane. Once a stable plane of anesthesia was achieved, it was maintained with 0.7–1.0% isoflurane via inhalation (34). After cardiac perfusion with ice-cold normal saline, the whole brain was rapidly excised. The hippocampi were dissected on an ice-cold dissection platform, gently rinsed with ice-cold normal saline, and stored at −80 °C for subsequent analysis.

##### 2.6.2.3 Behavioral testing

The sucrose preference test (SPT) was used to assess stress-induced an hedonic behavior in the mice. (35). This behavioral paradigm for assessing depression-like phenotypes is predicated on the strong innate preference of mice for sucrose. Each mouse was given access to two bottles: one filled with plain water and the other filled with a 2% sucrose solution. After a 24-hour adaptation period, the positions of the two bottles were swapped every 12 hours to mitigate potential biases arising from side preference. To align with the nocturnal activity patterns of rodents, the experimental phase was conducted from 21:00 to 09:00. Bottle weights for water and sucrose solution were measured pre- and post-experiment. Fluid intake for each mouse was determined by calculating the weight loss of each bottle. Sucrose preference was calculated as the percentage of sucrose intake relative to total fluid intake.

The tail suspension test (TST) is a behavioral paradigm used to assess depression-like behaviors. It is based on the observation that mice, when suspended by their tails, initially attempt to escape but eventually discontinue active escape attempts and exhibit a characteristic immobile state interpreted as a measure of despair. One-third of the mouse tail was suspended in the tail suspension apparatus using adhesive tape to maintain the head approximately 20 cm above the ground. Mouse behavior was videotaped for 5 minutes. After each trial, the feces were removed, and the bottom of the suspension apparatus was disinfected with an ethanol-based solution. All the data were analyzed using EthoVision XT software from Noldus Information Technology.

The forced swimming test (FST) is a well-established paradigm for assessing depression-like behavior. After repeatedly unsuccessful attempts to escape within an inescapable water tank, mice gradually cease actively struggling and enter a state of floating immobility, which is indicative of depression-like despair behavior. The mice were individually placed in a transparent cylindrical container (approximately 20 cm in diameter and 27.5 cm in height). The water depth was maintained at approximately 20 cm to prevent the tail from providing support, and the water temperature was maintained between 23 and 25 °C. Each mouse underwent a 5-minute swimming trial. The water in the cylinder was replaced before each trial to eliminate olfactory interference. The behavior of the mice was videotaped from a lateral view. All the data were analyzed using EthoVision XT software from Noldus Information Technology.

##### 2.6.2.4 Western blotting analysis

Western blotting was employed to assess the expression levels of specific proteins in the mouse prefrontal cortex, namely, brain-derived neurotrophic factor (BDNF), postsynaptic density protein-95 (PSD-95), and mitophagy-related proteins, namely, PTEN-induced kinase 1 (PINK1), Parkin RBR E3 ubiquitin protein ligase (Parkin), microtubule-associated protein 1 light chain 3 beta (LC3B), and sequestosome 1 (SQSTM1/P62). After mouse brain tissue samples were collected, total protein was extracted using RIPA lysis buffer. The protein concentration was measured with a BCA protein quantification kit (Beyotime, China). Protein samples were heat-denatured at 100 °C. Equal amounts of protein were subsequently separated by 10% sodium dodecyl sulfate–polyacrylamide gel electrophoresis (SDS–PAGE) (Yamay, China). After electrophoretic separation, the proteins were transferred onto PVDF membranes and subsequently blocked with 5% skim milk for 1 h. The membranes were incubated with primary antibodies overnight at 4 °C. The primary antibodies and their working dilutions were as follows: anti-BDNF (recombinant rabbit monoclonal, 1:10,000; Huabio), anti-PSD95 (rabbit monoclonal, 1:2,000; Zenbio), anti-PINK1 (rabbit monoclonal, 1:2,000; Huabio), anti-Parkin (rabbit monoclonal, 1:1,000; ABMART), anti-LC3B (rabbit polyclonal, 1:3,000; Huabio), anti-P62 (rabbit monoclonal, 1:20,000; Huabio), and anti-GAPDH (rabbit monoclonal, 1:5,000; Zenbio). The membranes were subsequently washed with TBST and incubated with horseradish peroxidase (HRP)-conjugated secondary antibodies at room temperature for 1 h. After treatment with ECL luminescent substrate (Proteintech, China), chemiluminescence was detected using a ChemiScope imaging system (Shanghai Junsci Scientific Instrument Co., Ltd., China). The grayscale intensity of the protein bands was quantified using ImageJ software. GAPDH served as the internal reference protein for normalization. Relative expression levels of target proteins were then compared across groups.

##### 2.6.2.5 Quantitative polymerase chain reaction

*CCT3*, *EEF2*, *EIF3I*, and *RPS5* mRNA expression levels were primarily quantified. Following the manufacturer’s instructions, total RNA was extracted from the hippocampal tissue homogenates using TRIzol reagent (MagZol Reagent, China) and a chloroform substitute (Buffer BCP, China). Reverse transcription was carried out using Hifair III 1st Strand cDNA Synthesis SuperMix for qPCR (China) on a ProFlex thermal cycler. Quantitative real−time PCR (qPCR) was performed on a SLAN−96P Real−Time PCR System using SYBR Green Master Mix and gene−specific primers (Sangon Biotech, China), with 20 ng of cDNA used per reaction. The thermal cycling conditions were as follows: initial denaturation at 95 °C for 2 min, followed by 40 amplification cycles of 95 °C for 10 s and 60 °C for 30 s. All genes were analyzed in three technical replicates. Relative quantification was performed, and the data were normalized to those of β−actin, which served as the internal reference gene. The sequences of the primers used for qPCR in this study are listed in **Table 1**.

**Table 1:** Sequences of primers used for the qPCR analysis.

Gene	Direction	Primer sequences
*EEF2*	Forward Reverse	5’-AGTGTCCTGAGCAAGTGGTG-3’ 5’-AGATCAGCGGTGAAGCCAAA-3’
*CCT3*	Forward Reverse	5’-TCCTCCTTGGCATGCAACAT-3’ 5’-ATGCCCCCGGGTATCTTTTC-3’
*RPS5*	Forward Reverse	5’-TTCTAAGGCACATCTGGGGG-3’ 5’-CTTCCCACTCAGTCATCTCGG-3’
*EIF3I*	Forward Reverse	5’-TTCCGGTCCCACTCACATTG-3’ 5’-CCACACGTTGACGATAGGGT-3’

### 2.7 Statistical analysis

The mice were randomly assigned to experimental groups, while the outcome assessors remained blinded to the group assignments. Statistical analyses were performed using SPSS v. 29.0 (IBM Corp., Armonk, NY, USA) and GraphPad Prism v. 10.1.2 (GraphPad Software, San Diego, CA, USA). An independent two-sample t test was used to compare the two groups. All the data are presented as the mean ± standard deviation (SD). *P* < 0.05 was considered to indicate statistical significance.

## 3 Results

### 3.1 Identification of DEGs in MDD patients versus healthy controls

**Figure 1:**
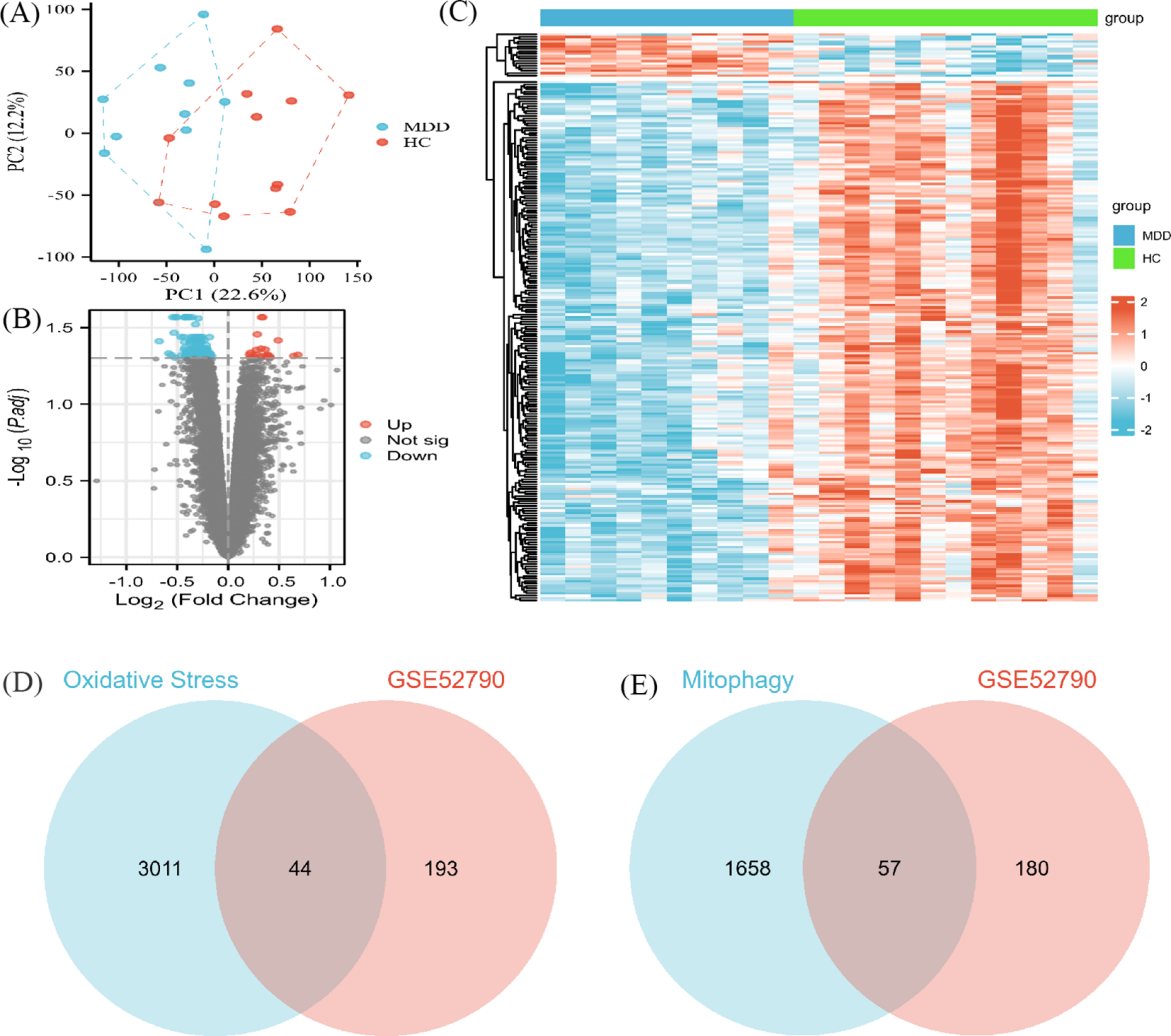
Analysis of differentially expressed genes (DEGs) in MDD. **(A)** Principal component analysis (PCA) of MDD (n=10) and HC (n=12) samples, using DEG expression profiles, revealed distinct separation between the groups, which accounted for 22.6% and 12.2% of the variance in PC1 and PC2, respectively. **(B)** Volcano plot illustrating differentially expressed genes (DEGs). Genes satisfying the thresholds of |log2(fold change)| > 1 and adjusted *P* value < 0.05 were considered significantly differentially expressed. Red dots indicate significantly upregulated genes, blue dots denote significantly downregulated genes, and gray dots represent nonsignificant genes. **(C)** Heatmap displaying the significant features and clustering analysis of MDD patients and healthy controls. **(D)** Venn diagram showing the intersection of DEGs and oxidative stress-related genes. **(E)** Venn diagram of DEGs intersecting with mitophagy-related genes.

The gene expression dataset GSE52790, comprising 10 patients with MDD and 12 healthy controls, was downloaded from the GEO database for comprehensive gene expression analysis. Principal component analysis (PCA) of the dataset revealed that PC1 and PC2 accounted for 22.6% and 12.2% of the variance, respectively, indicating a distinct separation between the two groups and validating the suitability for subsequent differential expression analysis (**Figure 1A**). On the basis of the criteria of adjusted *P* value < 0.05 and |Log2FC| > 1, a total of 239 differentially expressed genes (DEGs) were identified in the comparison between the MDD and control groups. After excluding 4 long noncoding RNAs (lncRNAs), we identified 235 DEGs (data summarized in **Table 2**), with 214 downregulated and 21 upregulated (**Figures 1B, C**). We retrieved two gene sets comprising 1716 mitophagy-related genes and 3056 oxidative stress-related genes from the GeneCards database. Intersecting these gene sets with the DEGs revealed 44 overlapping DEGs associated with oxidative stress and 57 linked to mitophagy (**Figures 1D, E**).

**Table 2:** Differentially expressed genes (DEGs) identified from the comparison of MDD versus HC in the GEO dataset GSE52790, listing their detailed information.

Rank	ID	Gene Symbol	logFC	Adjusted P-value	P-value	Regulation
1	TC0501312	HNRNPA0	-5.01E-01	0.0271	2.56E-06	Down
2	TC1900927	ZNF543	-4.21E-01	0.0271	3.73E-06	Down
3	TC0102490	RPSAP19	-4.21E-01	0.0271	4.09E-06	Down
4	TC2000223	ERGIC3	-3.96E-01	0.0271	4.27E-06	Down
5	TC1101262	FANCF	-5.19E-01	0.0271	4.66E-06	Down
6	TC1000589	LOC143188	3.29E-01	0.0271	5.06E-06	Up
7	TC0901453	EDF1	-3.80E-01	0.0271	5.12E-06	Down
8	TC0900914	TOMM5	-3.52E-01	0.0271	5.21E-06	Down
9	TC1100448	TMEM109	-4.25E-01	0.0271	5.58E-06	Down
10	TC1000349	ADK	-5.42E-01	0.0271	6.81E-06	Down
11	TC1701013	RPL26	-2.94E-01	0.0275	7.80E-06	Down
12	TC1901046	EEF2	-3.20E-01	0.0301	1.12E-05	Down
13	TC1900960	RPS5	-5.31E-01	0.0342	1.71E-05	Down
14	TC0100801	ATXN7L2	4.78E-02	0.467	2.19E-01	Up
15	TC0101525	IARS2	-3.58E-01	0.0363	2.08E-05	Down
16	TC1101609	C11orf24	-1.79E-01	0.0856	4.07E-03	Down
17	TC0301409	RPL24	-3.13E-01	0.0364	2.37E-05	Down
18	TC0103366	TOMM20	-3.11E-01	0.0364	2.45E-05	Down
19	TC0601169	HLA-DOA	-4.32E-01	0.0364	2.58E-05	Down
20	TC1000414	MINPP1	-4.62E-01	0.0364	2.74E-05	Down
21	TC0601554	TSPYL1	-2.78E-01	0.0364	2.86E-05	Down
22	TC0601181	RPL12P1	-2.75E-01	0.0994	6.18E-03	Down
23	TC0800014	AGPAT5	-2.79E-01	0.0364	3.17E-05	Down
24	TC1900299	DDA1	-1.83E-01	0.0365	3.25E-05	Down
25	TC1000605	TRUB1	-2.77E-01	0.0371	3.41E-05	Down
26	TC1100601	AIP	-3.26E-01	0.038	3.69E-05	Down
27	TC0X00482	PRPS1	-3.65E-01	0.0383	3.84E-05	Down
28	TC1900957	TRIM28	-2.67E-01	0.0383	4.05E-05	Down
29	TC1501067	LOC100129840	4.91E-01	0.0383	4.13E-05	Up
30	TC2000472	PRPF6	-2.35E-01	0.0383	4.14E-05	Down
31	TC2200649	MFNG	-3.48E-01	0.0383	4.21E-05	Down
32	TC1700614	NOG	-6.76E-01	0.0388	4.65E-05	Down
33	TC1900519	MED29	-2.68E-01	0.0388	4.75E-05	Down
34	TC0700057	C7orf26	-3.12E-01	0.0393	5.10E-05	Down
35	TC1600637	KLHL36	-2.92E-01	0.0393	5.30E-05	Down
36	TC0701651	AKR1B1	-3.69E-01	0.0393	5.91E-05	Down
37	TC1900675	GLTSCR2	-3.02E-01	0.0393	6.12E-05	Down
38	TC1300043	CDK8	-2.95E-01	0.0393	6.30E-05	Down
39	TC1100910	TRAPPC4	-3.14E-01	0.0393	6.41E-05	Down
40	TC1600524	CTCF	-2.67E-01	0.0393	6.42E-05	Down
41	TC1601144	GOT2	-4.28E-01	0.0393	6.43E-05	Down
42	TC0800981	ARMC1	-3.01E-01	0.0395	6.51E-05	Down
43	TC0900081	RRAGA	-3.72E-01	0.0399	6.69E-05	Down
44	TC2200265	EIF3L	-3.20E-01	0.0399	6.74E-05	Down
45	TC0700056	ZDHHC4	-2.14E-01	0.0411	7.25E-05	Down
46	TC1901497	FBL	-4.24E-01	0.0427	7.73E-05	Down
47	TC0200906	UBE2E3	-2.48E-01	0.0428	7.86E-05	Down
48	TC2200298	ADSL	-3.26E-01	0.0428	8.16E-05	Down
49	TC2200633	EIF3D	-2.77E-01	0.0428	8.39E-05	Down
50	TC0701056	FAM126A	-3.28E-01	0.0434	8.86E-05	Down
51	TC0200342	CCT7	-3.67E-01	0.0434	9.02E-05	Down
52	TC0300643	PCCB	-3.42E-01	0.0434	9.15E-05	Down
53	TC1701421	UBTF	-2.12E-01	0.0434	9.29E-05	Down
54	TC1200721	PLBD2	-2.11E-01	0.0434	9.57E-05	Down
55	TC0102865	MRPL24	-2.95E-01	0.0434	9.84E-05	Down
56	TC0100357	EIF3I	-2.79E-01	0.0434	9.89E-05	Down
57	TC0700409	RHBDD2	-2.62E-01	0.0434	9.91E-05	Down
58	TC1501220	Q8N2D2_HUMAN	3.25E-01	0.0434	1.00E-04	Up
59	TC1900233	C19orf53	-3.49E-01	0.0434	1.01E-04	Down
60	TC1401160	AHNAK2	3.63E-01	0.0439	1.03E-04	Up
61	TC0600358	TBC1D22B	-2.71E-01	0.044	1.04E-04	Down
62	TC0401444	Q8NEQ2_HUMAN	2.76E-01	0.0442	1.05E-04	Up
63	TC0101587	RNF187	-2.23E-01	0.0442	1.06E-04	Down
64	TC0200150	LBH	-4.31E-01	0.0442	1.08E-04	Down
65	TC1701169	ALDOC	-3.10E-01	0.0442	1.09E-04	Down
66	TC2200569	NIPSNAP1	-2.41E-01	0.0442	1.10E-04	Down
67	TC0201085	RPL37A	-2.50E-01	0.0442	1.12E-04	Down
68	TC0100257	PITHD1	-3.41E-01	0.0442	1.13E-04	Down
69	TC1901211	C19orf43	-2.09E-01	0.0442	1.14E-04	Down
70	TC1600553	VPS4A	-2.90E-01	0.0442	1.14E-04	Down
71	TC1300103	RGCC	-2.17E-01	0.0443	1.17E-04	Down
72	TC0X00890	UXT	-2.99E-01	0.0452	1.22E-04	Down
73	TC0X00322	IGBP1P2	-2.96E-01	0.0452	1.22E-04	Down
74	TC1100931	TBCEL	-3.40E-01	0.0452	1.25E-04	Down
75	TC2200183	UQCR10	-3.94E-01	0.0452	1.26E-04	Down
76	TC0100373	ZNF362	-3.05E-01	0.0452	1.29E-04	Down
77	TC0X01189	RPL39	-2.98E-01	0.0452	1.32E-04	Down
78	TC2100162	RRP1B	-2.85E-01	0.0452	1.38E-04	Down
79	TC0701317	BAZ1B	-2.55E-01	0.0452	1.40E-04	Down
80	TC1901155	EIF3G	-3.27E-01	0.0452	1.43E-04	Down
81	TC0201550	CCT4	-4.06E-01	0.0452	1.44E-04	Down
82	TC1700955	C1QBP	-2.68E-01	0.0452	1.46E-04	Down
83	TC0301876	PPP1R2	-3.13E-01	0.0452	1.48E-04	Down
84	TC1200217	MRPS35	-3.30E-01	0.0452	1.48E-04	Down
85	TC1701607	SMARCD2	-2.29E-01	0.0452	1.48E-04	Down
86	TC1900010	CDC34	-4.02E-01	0.0452	1.49E-04	Down
87	TC2000405	RBM38	-3.76E-01	0.0456	1.51E-04	Down
88	TC0102852	CCT3	-2.87E-01	0.0458	1.53E-04	Down
89	TC2200764	CERK	-2.42E-01	0.0458	1.54E-04	Down
90	TC0200738	R3HDM1	-2.49E-01	0.0458	1.54E-04	Down
91	TC1101634	NUMA1	-2.57E-01	0.0458	1.55E-04	Down
92	TC1201252	NPFF	2.21E-01	0.0461	1.59E-04	Up
93	TC0100169	PLEKHM2	-2.57E-01	0.0461	1.60E-04	Down
94	TC0301199	QARS	-2.85E-01	0.0461	1.62E-04	Down
95	TC2100072	SOD1	-2.36E-01	0.0461	1.64E-04	Down
96	TC1100004	RIC8A	-1.87E-01	0.0461	1.64E-04	Down
97	TC1700180	ALKBH5	-3.43E-01	0.0461	1.67E-04	Down
98	TC0400303	DCK	-5.83E-01	0.0466	1.70E-04	Down
99	TC0601274	MEA1	-2.89E-01	0.0466	1.75E-04	Down
100	TC0X00595	HTATSF1	-2.77E-01	0.0467	1.79E-04	Down
101	TC1501037	PDCD7	-1.77E-01	0.0467	1.80E-04	Down
102	TC1501183	RPS17	-2.82E-01	0.047	1.87E-04	Down
103	TC0600398	KLHDC3	-3.55E-01	0.047	1.88E-04	Down
104	TC0800078	FDFT1	-2.53E-01	0.047	1.89E-04	Down
105	TC0700086	BZW2	-2.95E-01	0.047	1.90E-04	Down
106	TC1601125	CIAPIN1	-3.37E-01	0.047	1.91E-04	Down
107	TC1200766	ORAI1	-2.40E-01	0.047	1.91E-04	Down
108	TC0300247	DHX30	-2.18E-01	0.047	1.93E-04	Down
109	TC2000897	YTHDF1	-2.20E-01	0.047	1.94E-04	Down
110	TC1701004	VAMP2	-1.80E-01	0.047	1.97E-04	Down
111	TC1200693	FAM216A	-3.74E-01	0.047	1.97E-04	Down
112	TC2200233	MCM5	-2.47E-01	0.047	2.01E-04	Down
113	TC0100272	TMEM57	-2.63E-01	0.047	2.02E-04	Down
114	TC1900225	RAD23A	-2.22E-01	0.047	2.07E-04	Down
115	TC1900009	BSG	-3.01E-01	0.047	2.10E-04	Down
116	TC0501344	PFDN1	-2.70E-01	0.047	2.13E-04	Down
117	TC0300172	SLC25A38	-2.83E-01	0.047	2.14E-04	Down
118	TC1900507	EIF3K	-2.81E-01	0.047	2.14E-04	Down
119	TC1200514	KCNMB4	-3.74E-01	0.047	2.16E-04	Down
120	TC1700018	RPA1	-3.09E-01	0.047	2.19E-04	Down
121	TC1400817	FKBP3	-3.75E-01	0.047	2.20E-04	Down
122	TC1701283	RPL23	-2.97E-01	0.047	2.21E-04	Down
123	TC1700735	RPL38	-2.47E-01	0.047	2.22E-04	Down
124	TC0101225	DUSP12	-3.69E-01	0.047	2.22E-04	Down
125	TC1201565	HVCN1	-2.70E-01	0.047	2.23E-04	Down
126	TC1500738	UBE3A	-3.22E-01	0.047	2.24E-04	Down
127	TC0301023	RPL32	-2.22E-01	0.047	2.24E-04	Down
128	TC1200736	PEBP1	-3.07E-01	0.0471	2.26E-04	Down
129	TC0501514	KIAA1191	-2.82E-01	0.0472	2.28E-04	Down
130	TC0600666	RWDD1	-2.18E-01	0.0472	2.28E-04	Down
131	TC0400922	CORIN	2.10E-01	0.0473	2.30E-04	Up
132	TC1400370	ISCA2	-2.77E-01	0.0473	2.31E-04	Down
133	TC1200917	PHB2	-2.90E-01	0.0474	2.36E-04	Down
134	TC1901010	CSNK1G2-AS1	2.85E-01	0.0474	2.36E-04	Down
135	TC0901468	TMEM203	-2.13E-01	0.0474	2.39E-04	Down
136	TC0200135	GPN1	-2.94E-01	0.0474	2.41E-04	Down
137	TC1400794	MBIP	-3.54E-01	0.0474	2.45E-04	Down
138	TC2200124	SMARCB1	-1.95E-01	0.0474	2.48E-04	Down
139	TC2200464	THAP7	-1.83E-01	0.0474	2.48E-04	Down
140	TC0900732	EHMT1	-1.92E-01	0.0474	2.49E-04	Down
141	TC0900640	FUBP3	-2.80E-01	0.0474	2.52E-04	Down
142	TC0400217	DCUN1D4	-2.51E-01	0.0474	2.54E-04	Down
143	TC0X01369	BCAP31	-2.38E-01	0.0476	2.57E-04	Down
144	TC1901660	DBP	-1.99E-01	0.0476	2.57E-04	Down
145	TC0X01236	ENOX2	-2.00E-01	0.0476	2.58E-04	Down
146	TC1200903	MRPL51	-3.00E-01	0.0476	2.58E-04	Down
147	TC0300273	APEH	-2.66E-01	0.0476	2.59E-04	Down
148	TC1100954	Q8NH82_HUMAN	3.88E-01	0.0476	2.61E-04	Up
149	TC0X00204	FTSJ1	-2.21E-01	0.0476	2.61E-04	Down
150	TC0201381	PREB	-2.76E-01	0.0476	2.64E-04	Down
151	TC0200122	C2orf28	-3.35E-01	0.0477	2.68E-04	Down
152	TC0801072	ZFAND1	-3.48E-01	0.0477	2.68E-04	Down
153	TC2000432	RPS21	-2.30E-01	0.0477	2.68E-04	Down
154	TC1900563	CD79A	-5.59E-01	0.0478	2.70E-04	Down
155	TC1900151	HNRNPM	-2.33E-01	0.0478	2.72E-04	Down
156	TC0101163	CD1C	-4.57E-01	0.0479	2.74E-04	Down
157	TC0101809	RPL22	-2.38E-01	0.0479	2.75E-04	Down
158	TC0400595	MAB21L2	3.76E-01	0.048	2.78E-04	Up
159	TC1701785	P4HB	-2.90E-01	0.048	2.78E-04	Down
160	TC1200599	METAP2	-3.16E-01	0.048	2.79E-04	Down
161	TC0600067	PAK1IP1	-3.10E-01	0.0481	2.82E-04	Down
162	TC0600149	HIST1H2BD	-3.98E-01	0.0481	2.83E-04	Down
163	TC0900182	POLR1E	-4.18E-01	0.0481	2.83E-04	Down
164	TC0400976	NOA1	-4.17E-01	0.0481	2.84E-04	Down
165	TC0900726	COBRA1	-2.76E-01	0.0482	2.85E-04	Down
166	TC0200283	MDH1	-3.25E-01	0.0482	2.85E-04	Down
167	TC1500515	HMG20A	-2.21E-01	0.0483	2.87E-04	Down
168	TC0X01060	ITM2A	-3.55E-01	0.0484	2.92E-04	Down
169	TC1600750	HAGH	-1.63E-01	0.0484	2.93E-04	Down
170	TC0901026	Q6ZVS9_HUMAN	2.52E-01	0.0484	2.96E-04	Up
171	TC1200447	Q9UI61_HUMAN	4.04E-01	0.0484	2.96E-04	Up
172	TC1601202	CHTF8	-2.73E-01	0.0484	2.97E-04	Down
173	TC1601027	BCL7C	-1.85E-01	0.0484	3.01E-04	Down
174	TC0X00492	Q9P1I9_HUMAN	6.44E-01	0.0484	3.01E-04	Up
175	TC1601254	GLG1	-2.78E-01	0.0484	3.02E-04	Down
176	TC1400470	GLRX5	-2.53E-01	0.0484	3.04E-04	Down
177	TC0X00392	APOOL	-3.11E-01	0.0484	3.04E-04	Down
178	TC1900420	UBA2	-3.72E-01	0.0484	3.04E-04	Down
179	TC0102753	MRPL9	-2.86E-01	0.0484	3.05E-04	Down
180	TC1101461	DDB1	-2.86E-01	0.0484	3.06E-04	Down
181	TC2200318	ACO2	-2.91E-01	0.0484	3.08E-04	Down
182	TC0X00559	UTP14A	-3.19E-01	0.0484	3.09E-04	Down
183	TC0900415	HABP4	-2.69E-01	0.0485	3.13E-04	Down
184	TC2200566	AP1B1	-2.10E-01	0.0486	3.16E-04	Down
185	TC0500485	CAMLG	-3.87E-01	0.0486	3.17E-04	Down
186	TC1200497	DYRK2	-2.47E-01	0.0487	3.21E-04	Down
187	TC1900284	AP1M1	-2.62E-01	0.0487	3.23E-04	Down
188	TC0X00870	FUNDC1	-2.99E-01	0.0487	3.23E-04	Down
189	TC0901301	GSN	3.28E-01	0.0487	3.26E-04	Up
190	TC1101484	HNRNPUL2	-2.18E-01	0.0487	3.28E-04	Down
191	TC0102793	ILF2	-2.85E-01	0.0487	3.28E-04	Down
192	TC0X00883	NDUFB11	-3.00E-01	0.0487	3.29E-04	Down
193	TC2200033	MRPL40	-2.73E-01	0.0487	3.29E-04	Down
194	TC1300049	GTF3A	-2.50E-01	0.0487	3.30E-04	Down
195	TC0102079	MARCKSL1	-1.98E-01	0.0487	3.33E-04	Down
196	TC1300183	TDRD3	-2.22E-01	0.0487	3.34E-04	Down
197	TC0300601	C3orf37	-2.58E-01	0.0487	3.34E-04	Down
198	TC1900450	COX6B1	-3.99E-01	0.0487	3.35E-04	Down
199	TC1501175	RPS17	-3.00E-01	0.0487	3.36E-04	Down
200	TC17r00049	P4HB	-2.79E-01	0.0492	3.40E-04	Down
201	TC1701041	ELAC2	-2.58E-01	0.0492	3.44E-04	Down
202	TC0102244	FAF1	-1.99E-01	0.0492	3.44E-04	Down
203	TC1700425	PSMD3	-2.50E-01	0.0492	3.47E-04	Down
204	TC0102047	DNAJC8	-3.54E-01	0.0492	3.48E-04	Down
205	TC0901431	CAMSAP1	-1.84E-01	0.0492	3.49E-04	Down
206	TC0202313	AAMP	-1.93E-01	0.0492	3.50E-04	Down
207	TC1701809	WDR45L	-2.26E-01	0.0492	3.51E-04	Down
208	TC0500482	PHF15	-2.68E-01	0.0492	3.51E-04	Down
209	TC1001138	RNLS	-1.76E-01	0.0492	3.52E-04	Down
210	TC0501442	NP10_HUMAN	3.74E-01	0.0492	3.52E-04	Up
211	TC1900991	POLR2E	-3.74E-01	0.0492	3.53E-04	Down
212	TC0800620	MAF1	-2.54E-01	0.0492	3.53E-04	Down
213	TC0300173	RPSAP9	-3.03E-01	0.0492	3.54E-04	Down
214	TC0103425	TFB2M	-3.52E-01	0.0494	3.58E-04	Down
215	TC0900425	ANP32B	-2.87E-01	0.0495	3.64E-04	Down
216	TC1600557	CYB5B	-3.39E-01	0.0495	3.64E-04	Down
217	TC0200444	RPIA	-2.36E-01	0.0495	3.65E-04	Down
218	TC0500265	BTF3	-2.51E-01	0.0495	3.65E-04	Down
219	TC1701437	DCAKD	-2.65E-01	0.0495	3.66E-04	Down
220	TC1701369	ACLY	-2.75E-01	0.0495	3.69E-04	Down
221	TC1600967	NUPR1	2.18E-01	0.0495	3.71E-04	Up
222	TC0701527	ORC5	-3.07E-01	0.0496	3.76E-04	Down
223	TC1701622	Q8N0T0_HUMAN	2.44E-01	0.0496	3.76E-04	Up
224	TC0100301	NUDC	-2.48E-01	0.0496	3.86E-04	Down
225	TC2200258	LGALS1	-4.19E-01	0.0496	3.87E-04	Down
226	TC0800444	PTDSS1	-2.99E-01	0.0496	3.87E-04	Down
227	TC1101462	CYBASC3	-2.45E-01	0.0496	3.88E-04	Down
228	TC0X00186	USP11	-2.69E-01	0.0496	3.88E-04	Down
229	TC0600856	MRPL18	-2.48E-01	0.0496	3.89E-04	Down
230	TC1600693	Q9H9U3_HUMAN	4.13E-01	0.0496	3.92E-04	Up
231	TC1101466	C11orf10	-1.93E-01	0.0496	3.93E-04	Down
232	TC0501281	ZCCHC10	-2.98E-01	0.0498	3.97E-04	Down
233	TC1200507	YEATS4	-5.19E-01	0.0498	4.00E-04	Down
234	TC0101003	HIST2H2AC	-3.80E-01	0.0498	4.02E-04	Down
235	TC1100129	SMPD1	-2.34E-01	0.0498	4.02E-04	Down

The full dataset is available in the **Supplementary Material**.

### 3.2 Functional enrichment analysis of DEGs associated with oxidative stress and mitophagy

**Figure 2:**
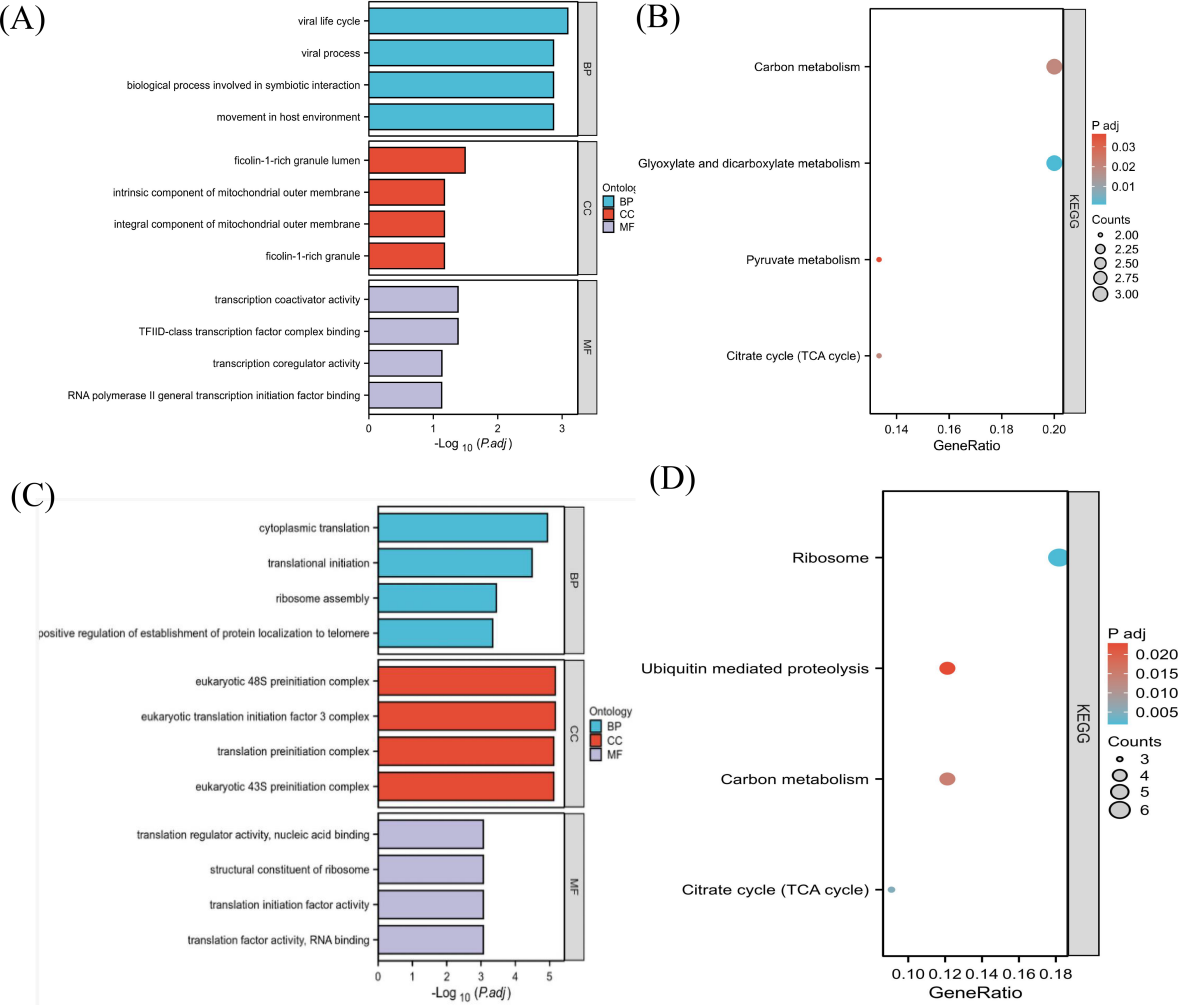
Identification and functional characterization of oxidative stress- and mitophagy-related genes. **(A)** GO enrichment analysis of 44 oxidative stress-related genes (DEGs). The functional annotation of the screened genes was performed using GO enrichment analysis. GO analysis categorizes gene functions into three major categories: biological process (BP), cellular component (CC), and molecular function (MF). Terms with an adjusted *P* value (*P.adj*) < 0.05 were considered significantly enriched. The bar chart displays the top four significantly enriched terms ranked by enrichment significance in each category. **(B)** KEGG pathway enrichment analysis revealed key pathways associated with 44 genes related to oxidative stress (DEGs) (*P.adj* < 0.05). The top four significantly enriched pathways are presented. **(C)** GO enrichment analysis of 57 DEGs related to mitophagy. The biological functions of the identified genes were annotated using GO analysis, a method that categorizes genes into three domains: biological process (BP), cellular component (CC), and molecular function (MF). Terms with an adjusted *P* value (*P.adj*) < 0.05 were considered significantly enriched. The bar chart illustrates the top four most significantly enriched terms for each category. **(D)** KEGG pathway enrichment analysis revealed key pathways associated with 57 mitophagy-related differentially expressed genes (DEGs) (*P.adj* < 0.05). The top four most highly enriched pathways are presented.

Intersecting oxidative stress-related genes with differentially expressed genes (DEGs) identified 44 genes (**Figure 1D**). GO enrichment analysis was conducted to elucidate functional attributes across three domains: biological process (BP), cellular component (CC), and molecular function (MF). The BP terms were predominantly associated with host–pathogen interaction, symbiosis, and microenvironment regulation, suggesting that these overlapping genes may maintain homeostasis of the neural microenvironment by modulating symbiotic interactions. The CC module is predominantly linked to protein secretion pathways, integral outer mitochondrial membrane proteins, and functional core complexes, underscoring their roles in subcellular localization and mitochondrial function. The MF module is significantly enriched for terms related to the assembly and regulation of transcription initiation complexes as well as transcriptional coregulation, indicating that these genes likely function as hubs in the transcriptional regulatory network to orchestrate the fine-tuning of gene expression across multiple signaling pathways. KEGG pathway enrichment analysis revealed significant enrichment of the 44 overlapping genes involved in carbon metabolism, oxalic acid and dicarboxylic acid metabolism, pyruvate metabolism, and the tricarboxylic acid (TCA) cycle (**Figures 2A, B**). These findings indicate that the identified overlapping genes are involved in the reprogramming of cellular energy metabolism, offering molecular support for the hypothesis linking energy metabolism disorders to depression.

The subsequent intersection of mitophagy-related genes and DEGs revealed 57 common genes (**Figure 1E**). GO enrichment analysis revealed that the biological process (BP) category was significantly enriched in processes associated with protein synthesis and quality control, as well as genomic stability maintenance, which are potentially related to the cellular stress response and global regulation of gene expression. The cellular component (CC) category was significantly enriched in cytoplasmic translation machinery and nuclear transcription machinery, highlighting the core subcellular structures mediating the flow of genetic information (DNA→RNA→protein). The molecular function (MF) category was enriched for functions involving transcriptional regulation and modulation of translational efficiency, suggesting that these genes play a central role in maintaining gene expression homeostasis. KEGG pathway enrichment analysis revealed that the 57 overlapping genes were significantly enriched in the ribosome, ubiquitin-mediated proteolysis, and central carbon metabolism pathways (**Figures 2C, D**), suggesting their role in the maintenance of protein homeostasis and energy reprogramming.

### 3.3 PPI network analysis and hub gene screening

**Figure 3:**
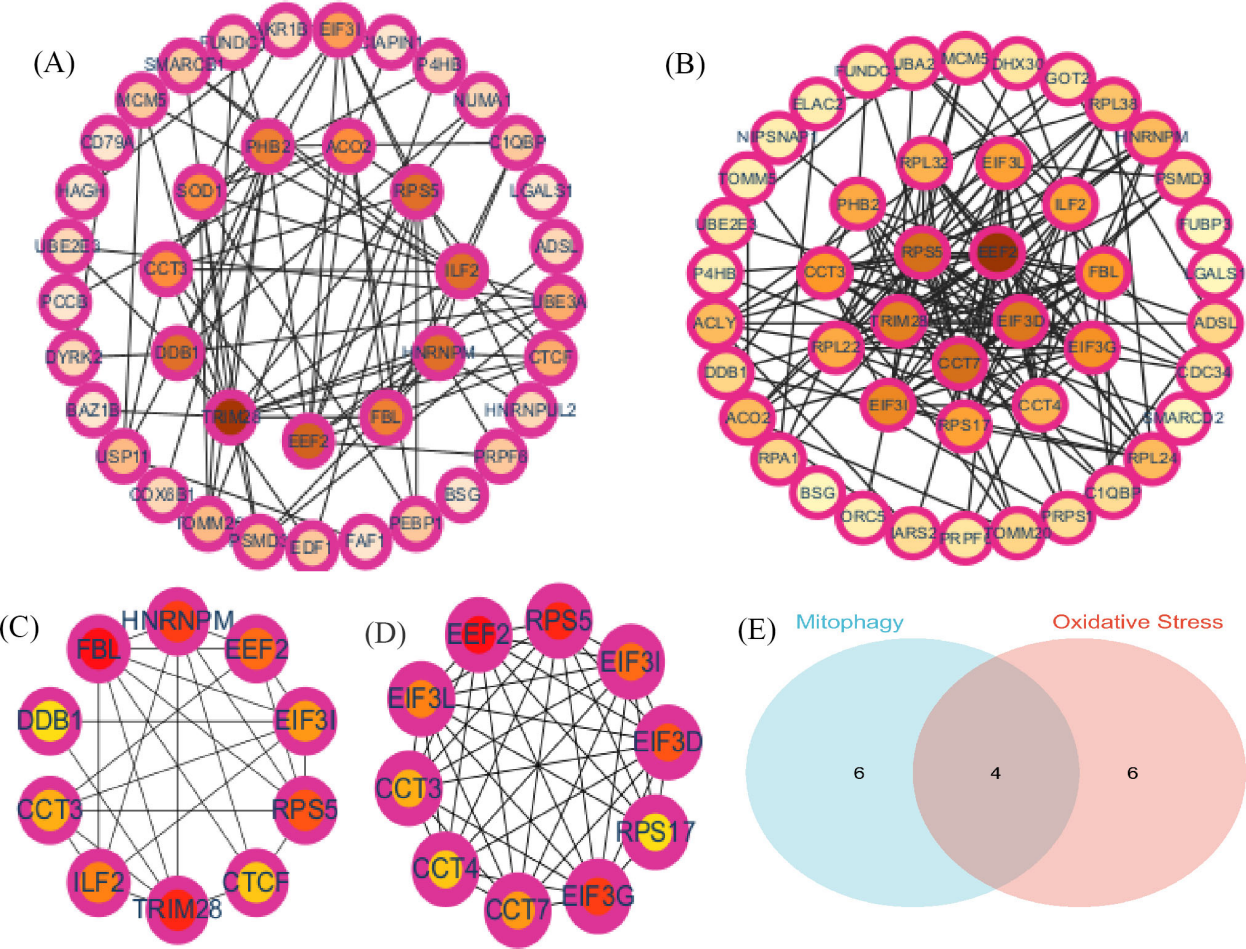
PPI network analysis of oxidative stress- and mitophagy-related DEGs. **(A)** The PPI network of oxidative stress-related DEGs. Nodes represent proteins corresponding to DEGs, while edges denote protein–protein interactions that are either experimentally validated or computationally predicted. **(B)** The PPI network of mitophagy-related DEGs. Nodes represent proteins encoded by DEGs, while edges represent protein–protein interactions. **(C)** Using the MCC algorithm, the top 10 hub genes were selected from the PPI network shown in Panel **(A)**. Hub genes are represented as larger nodes to denote their central importance. **(D)** The MCC algorithm was used to identify the top 10 hub genes within the PPI network shown in Panel **(B)**. Hub genes are depicted as larger nodes, with their size highlighting their central importance. **(E)** Venn diagram depicting the intersection of the hub genes identified in Panels **(C, D)**, which highlights three key common genes (*EEF2*, *CCT3*, and *EIF3I*).

Protein–protein interaction (PPI) networks for DEGs associated with oxidative stress and mitophagy were independently constructed using the STRING 12.0 database, and distinct networks were generated and subsequently visualized using Cytoscape 3.10.3 (**Figures 3A, B**). The MCC algorithm within the CytoHubba plugin was employed to identify hub genes in each network. The top 10 hub genes among the identified oxidative stress-related DEGs were *FBL*, *TRIM28*, *HNRNPM*, *RPS5*, *EEF2*, *ILF2*, *EIF3I*, *CCT3*, *CTCF*, and *DDB1* (**Figure 3C**), all of which were downregulated. The top 10 hub genes among the mitophagy-related DEGs were *EEF2*, *RPS5*, *EIF3G*, *EIF3D*, *EIF3I*, *EIF3L*, *CCT7*, *CCT3*, *CCT4*, and *RPS17* (**Figure 3D**), all of which were downregulated. We identified four candidate genes (*EEF2*, *CCT3*, *EIF3I*, and *RPS5*) common to both the mitophagy and oxidative stress gene sets. (**Figure 3E**).

### 3.4 Validation of candidate gene expression

**Figure 4:**
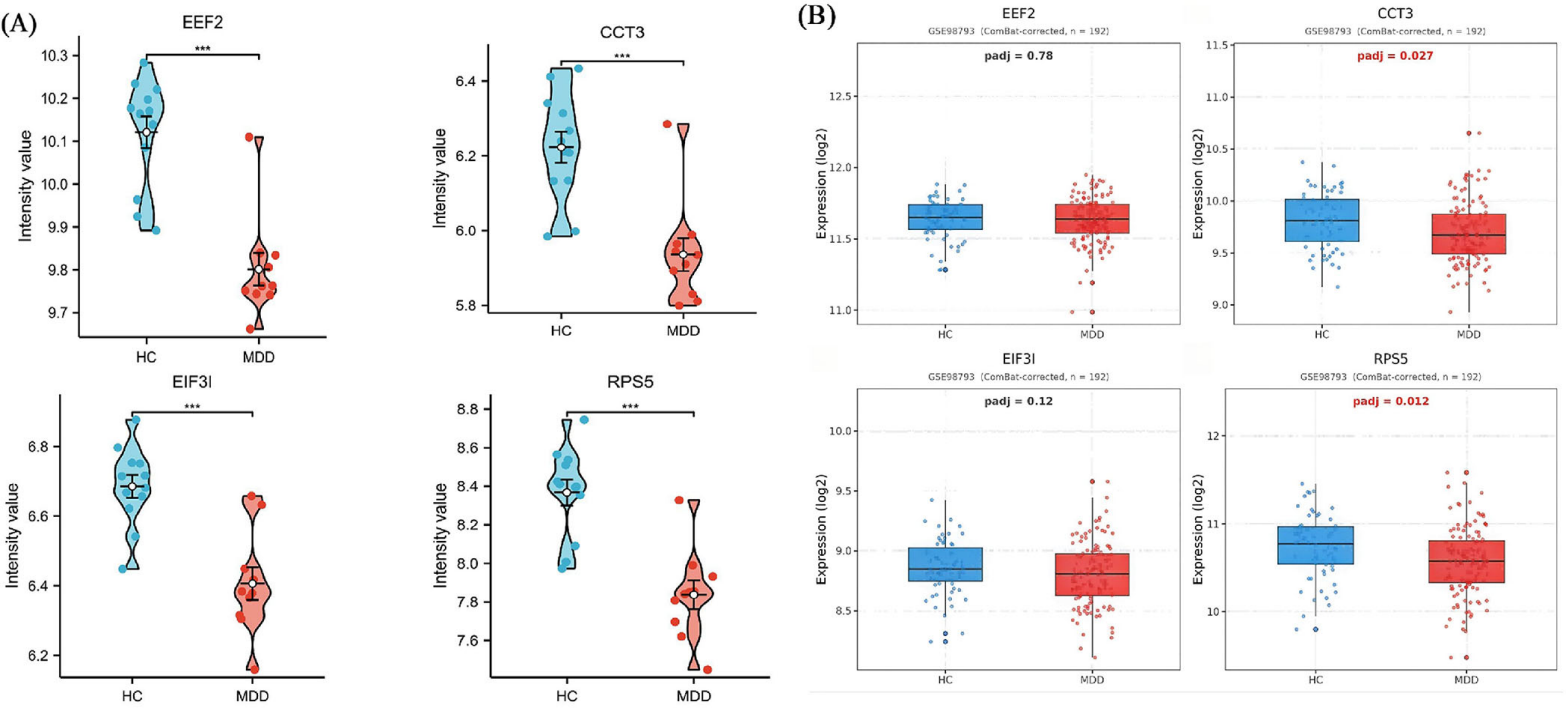
Expression of candidate genes. **(A)** All four candidate genes from the GSE52790 dataset were significantly downregulated in the MDD group. Specifically, *EEF2* (*Mann–Whitney U* = 116, ***P < 0.001) and *CCT3* (*Mann–Whitney U* = 111, ***P < 0.001) expression significantly decreased, whereas *EIF3I* (*Student’s t test*: *t* = -4.99, ***P < 0.001) and *RPS5* (*Student’s t test*: *t* = -5.30, ***P < 0.001) expression significantly decreased. **(B)** Comparison of the expression levels of four candidate genes (*EEF2*, *CCT3*, *EIF3I*, and *RPS5*) between healthy controls (HC) and individuals with MDD in the independent validation dataset GSE98793 following ComBat batch correction. The y-axis of the box plot represents log2-transformed gene expression values, whereas the x-axis indicates the study groups (HC or MDD). *EEF2* expression did not differ significantly between the HC and MDD groups (*P.adj* = 0.78). *CCT3* expression was significantly lower in the MDD group than in the HC group (*P.adj* = 0.027). *EIF3I* expression levels did not differ significantly between the HC and MDD groups (*P.adj* = 0.12). *RPS5* expression was significantly lower in the MDD group than in the HC group (*P.adj* = 0.012).

Four candidate genes were identified within the GSE52790 dataset through data mining analysis. The differential expression of these genes between the two groups was subsequently examined using the Xiantao platform. The expression levels of *EEF2*, *CCT3*, *EIF3I*, and *RPS5* were significantly lower in MDD patients than in controls (**Figure 4A**). To verify the generalizability of the aforementioned differential expression patterns, we observed that the four candidate genes exhibited a consistent downregulation trend in the validation dataset GSE98793. Following Benjamini–Hochberg (BH) correction for multiple testing (m=4, applied exclusively to the candidate gene set), the adjusted P values were as follows: *EEF2* (*P.adj* = 0.78), *EIF3I* (*P.adj* = 0.12), *CCT3* (*P.adj* = 0.027), and *RPS5* (*P.adj* = 0.012). Notably, compared with those in the healthy control group, the expression levels of CCT3 and RPS5 were significantly lower. The candidate genes exhibited consistent directions of expression changes between the training set and the validation set, despite the absence of perfect concordance. Nevertheless, their downregulated expression pattern suggests a stable molecular signature of MDD. (**Figure 4B**).

### 3.5 Molecular characterization of candidate genes

**Figure 5:**
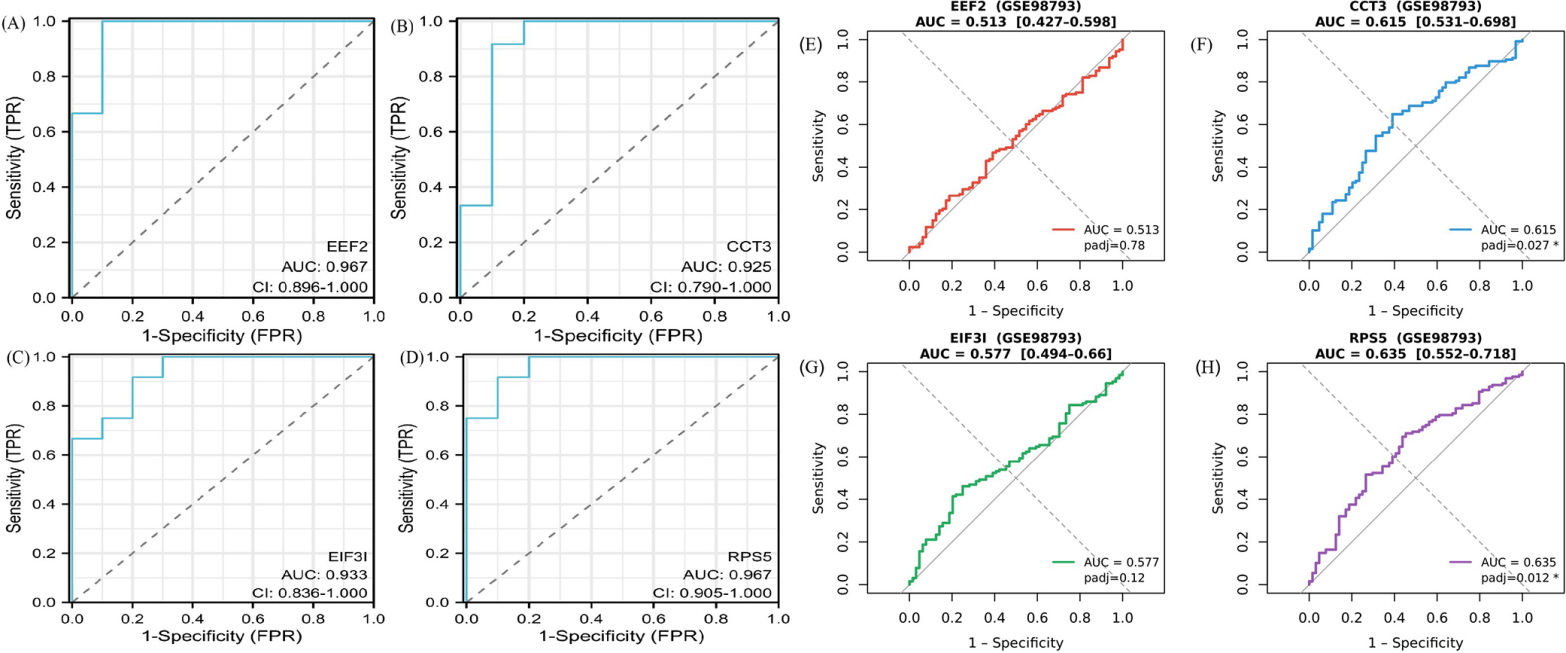
ROC curve analyses of candidate genes across the training and validation datasets. **(A–D)** ROC curves for the individual genes *EEF2*, *CCT3*, *EIF3I*, and *RPS5* in the training dataset GSE52790. The x-axis denotes the false positive rate (1-specificity), while the y-axis indicates the true positive rate (sensitivity). The diagonal line represents the reference line for the chance performance (AUC = 0.5). Each subplot displays the AUC values and 95% CIs for the corresponding gene. Notably, both *EEF2* and *RPS5* achieved an AUC value of 0.967, which may indicate a tendency toward overfitting. **(E–H)** ROC curves for the candidate genes in the independent validation dataset GSE98793. *CCT3* and *RPS5* were significantly different (*P.adj* < 0.05); however, their AUC values were 0.615 and 0.635, respectively, indicating modest diagnostic efficacy. In contrast, the differences in the expression levels of *EEF2* and *EIF3I* failed to reach statistical significance.

On the basis of the GSE52790 dataset, the area under the curve (AUC) values for (**Figure 5A**)*, CCT3* (**Figure 5B**)*, EIF3I* (**Figure 5C**) and *RPS5* (**Figure 5D**) were 0.967 (95% CI: 0.896–1.000), 0.925 (95% CI: 0.790–1.000), 0.933 (95% CI: 0.836–1.000), and 0.967 (95% CI: 0.905–1.000), respectively. In the validation dataset GSE98793, ROC curve analysis revealed AUC values of 0.513 (95% CI: 0.427–0.598) for *EEF2*(**Figure 5E**), 0.615 (95% CI: 0.531–0.698) for *CCT3*(**Figure 5F**), 0.577 (95% CI: 0.494–0.660) for *EIF3I*(**Figure 5G**), and 0.635 (95% CI: 0.552–0.718) for *RPS5* (**Figure 5H**). The AUC values of the four genes in the validation set did not meet the threshold required for clinically applicable diagnostic performance (AUC ≥ 0.7). Nevertheless, this screening approach retains exploratory value. The stable downregulation of the four genes in the training set is consistent with the energy metabolism hypothesis of major depressive disorder (MDD)—encompassing mitochondrial autophagy, oxidative stress, and protein quality control—suggesting their potential as promising candidate targets for subsequent mechanistic research. The sustained downregulation of *CCT3* and *RPS5* expression also suggests that these two genes may serve as potential molecular markers for MDD. Further validation is warranted in larger, more homogeneous cohorts using peripheral blood or cerebrospinal fluid samples. The AUC of the combined model on the training set was markedly high, indicating substantial overfitting and significantly impaired generalization performance.

### 3.6 Results of cell experiments

#### 3.6.1 Corticosterone treatment affected the viability of C8−D1A cells

**Figure 6:**
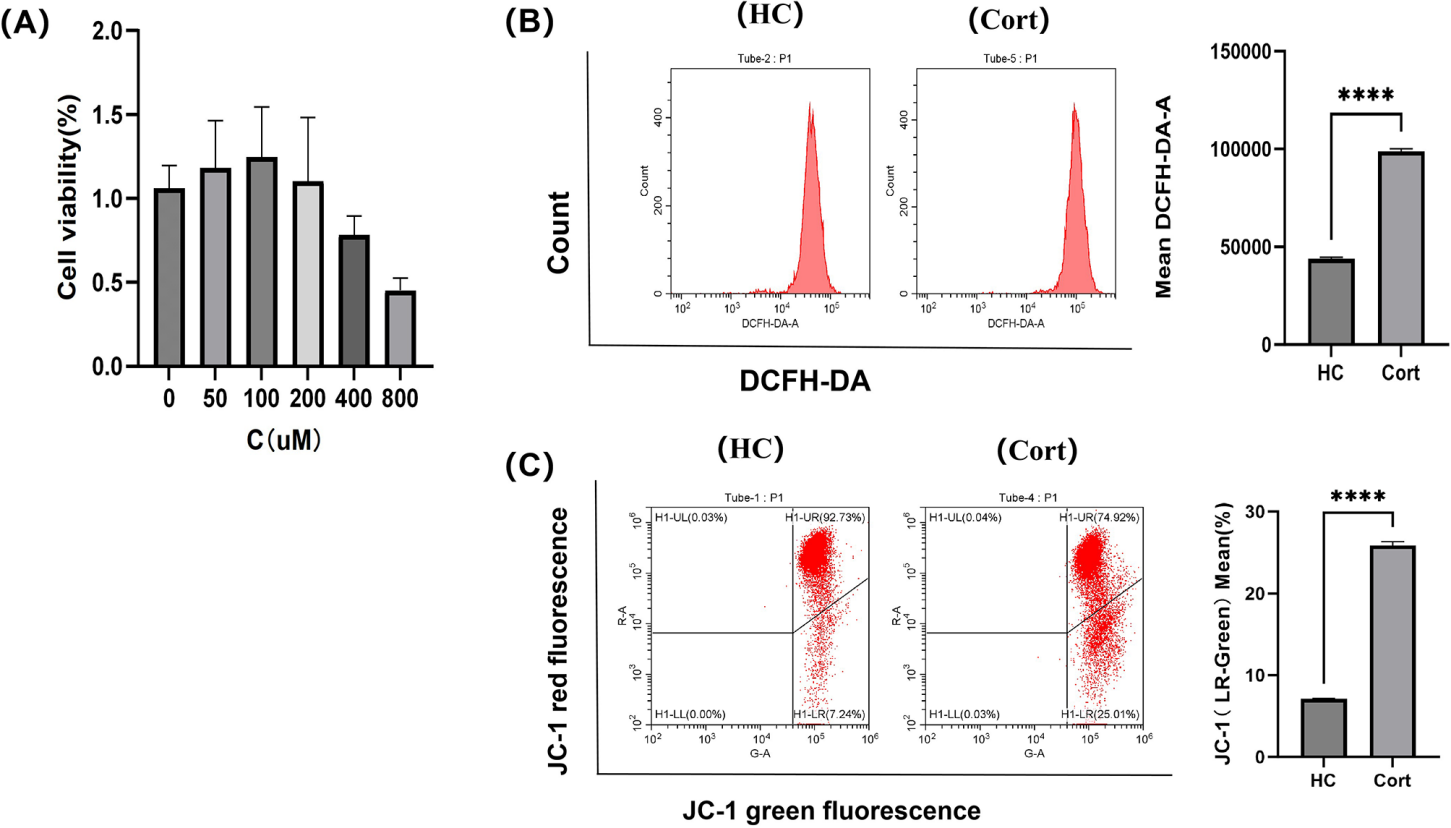
Effects of corticosterone on C8-D1A cell viability, ROS production, and mitochondrial function. **(A)** Cell viability following treatment with varying concentrations of corticosterone. At low concentrations (0–200 μM), cell viability was maintained at high levels. In contrast, cell viability decreased significantly at 400 μM, indicating that this higher concentration of corticosterone may negatively affect cell viability. **(B)** The two histograms in the left panel depict the distributions of intracellular DCFH-DA fluorescence intensity for the HC and cort-treated groups. The bar graph presented on the right compares the mean intracellular DCFH-DA fluorescence intensity across the two groups. The mean DCFH-DA fluorescence intensity in the Cort-treated group was significantly greater than that in the control group (****P<0.0001). **(C)** The two scatter plots on the left represent the HC group and the Cort-treated group. The H1-UR quadrant represents cells exhibiting high intensities of both green and red fluorescence. In contrast, the H1-LR quadrant denotes cells with high green but low red fluorescence intensity. The bar graph presented on the right compares the average mitochondrial membrane potential, quantified by the mean JC-1 green fluorescence intensity, between the control and Cort-treated groups. The results demonstrated that the mitochondrial membrane potential was significantly lower in the Cort-treated group than in the control group (****P < 0.0001).

Following exposure to corticosterone, cell viability decreased in a dose-dependent manner at concentrations ≥100 μmol/L, reaching approximately 70% at a concentration of 400 μmol/L (**Figure 6A**). Therefore, this concentration and the corresponding treatment duration were selected for subsequent experiments.

#### 3.6.2 Flow cytometric analysis of the corticosterone-induced increase in ROS generation and reduction in mitochondrial membrane potential in C8-D1A cells

Intracellular ROS levels were measured using the nonfluorescent probe DCFH-DA, which is oxidized to fluorescent DCF following oxidation by ROS (**Figure 6B**). Compared with untreated control cells, Corticosterone-treated C8-D1A cells exhibited significantly higher DCF fluorescence, with a mean fluorescence intensity (MFI) of 98740.83 ± 1367.05, indicating excessive corticosterone-induced ROS production.

Mitochondrial membrane potential (MMP) was measured by flow cytometry using a JC-1 fluorescent probe. JC-1 forms red fluorescent aggregates at high mitochondrial membrane potential, whereas it exists as green fluorescent monomers at lower potentials. The red-to-green fluorescence intensity ratio reflects the mitochondrial membrane potential. Compared with that in the control group, the JC−1 red/green fluorescence ratio was significantly lower in the corticosterone-treated C8-D1A cells. The percentage of depolarized cells in the LR quadrant increased to 25.89% ± 0.44% (**Figure 6C**), suggesting that corticosterone reduced the mitochondrial membrane potential, which is a hallmark of early mitochondrial damage.

### 3.7 Animal experimental results

**Figure 7:**
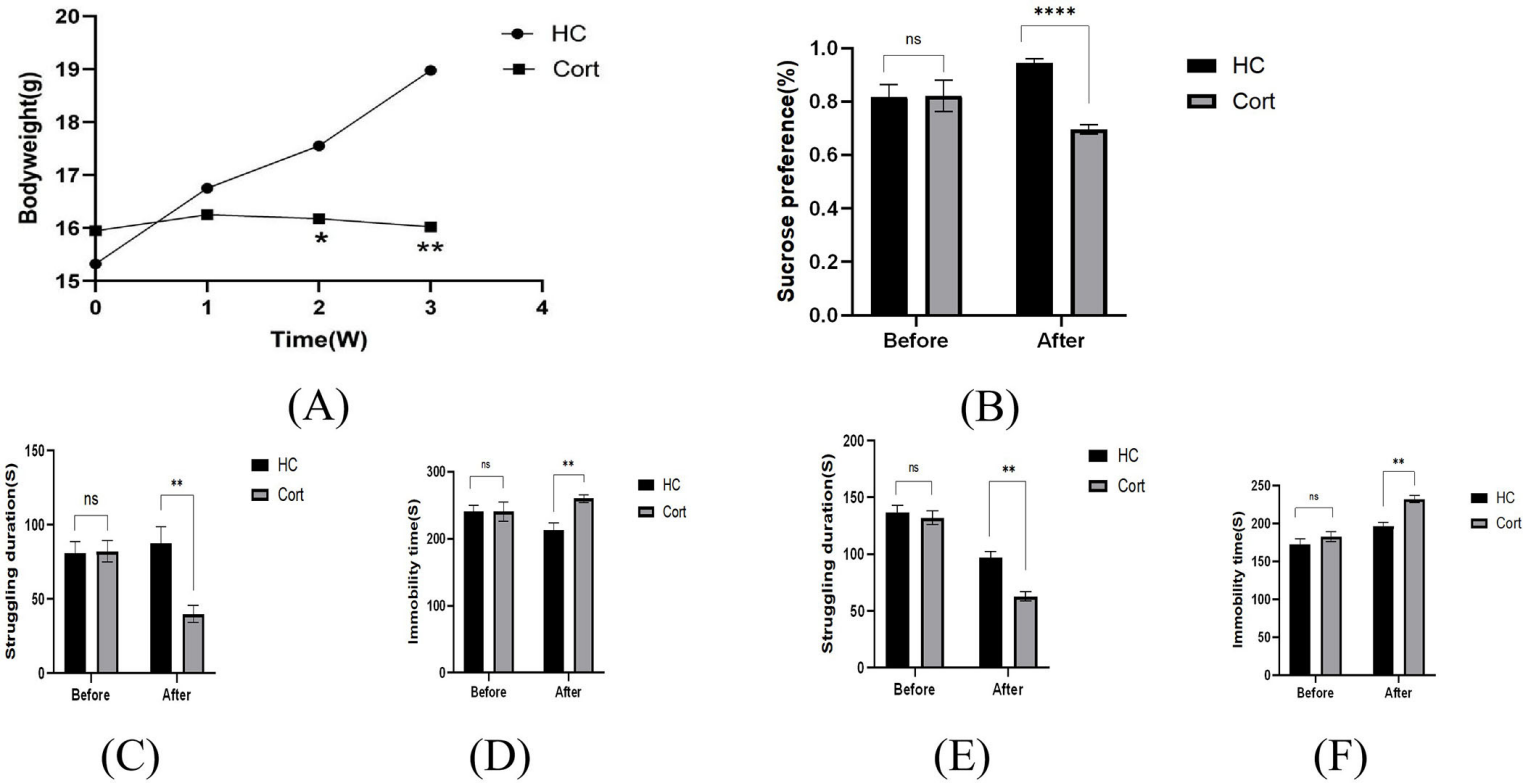
Alterations in the general health status and behavior of mice before and after model induction. **(A)** The body weights of the mice tended to decrease after stress exposure (*P < 0.05, **P < 0.005). **(B)** Sucrose preference in the SPT was compared between the experimental subjects and the healthy control group (ns *P*>0.05, ****P < 0.0001). **(C, D)** Duration of struggling and duration of immobility in the TST versus the healthy control group (ns *P*>0.05, **P < 0.005). **(E, F)** Struggling time and immobility time in the FST were compared to those in healthy controls (ns *P*>0.05, **P < 0.005).

In general, we found that the depression-model mice exhibited gradual and significant anorexia and weight loss, and their body weights were recorded at baseline (15.95 ± 0.70 g), 1 week (16.25 ± 0.56 g), 2 weeks (16.18 ± 0.14 g), and 3 weeks (16.03 ± 0.45 g). The body weight began to decrease at the second week (*P* < 0.05), and the reduction became more significant at the third week (*P* < 0.005) (**Figure 7A**).

**Figure 8:**
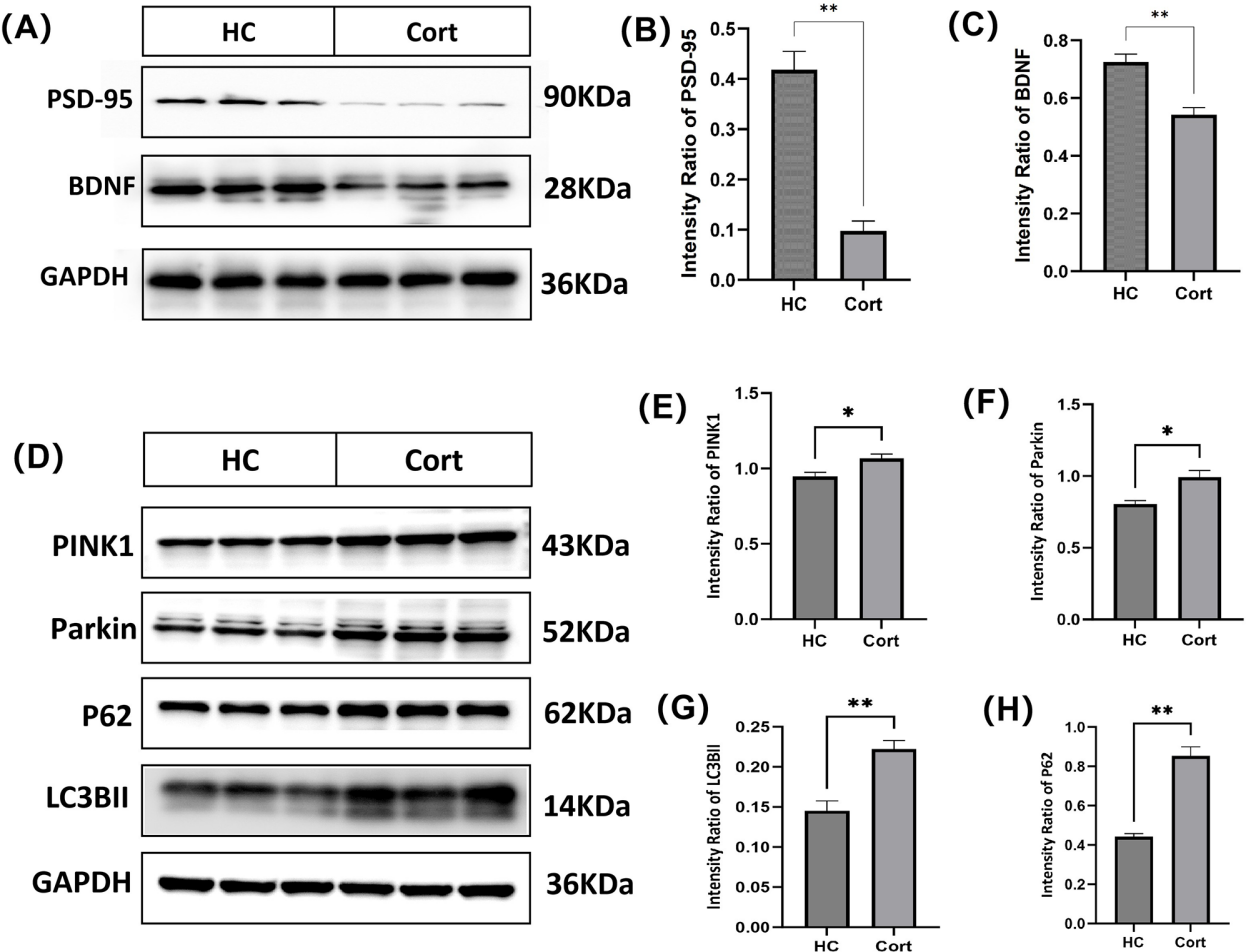
Differential expression of synaptic and mitophagy-related proteins was observed across the treatment groups. **(A–C))** The expression levels of the synapse-related proteins BDNF and PSD-95 were significantly decreased in the mice in the Cort-treated group (**P < 0.005). **(D–H)** The protein levels of the mitophagy-related proteins PINK1, Parkin, LC3BII, and P62 were significantly increased in the mice in the Cort-treated group (*P < 0.05, **P < 0.005).

All behavioral assessments were conducted in a double-blind manner both before and after the induction of the corticosterone model, with the results presented in **Figures 7B–F**. After 3 weeks of corticosterone exposure, the treated mice exhibited significant depression-like behaviors. Compared with that of the control group, the sucrose preference rate of the depression group in the SPT decreased significantly to 70 ± 18% (*P* < 0.0001) (**Figure 7B**). In the TST, the struggling duration of the depressed group was significantly shorter (39.87 ± 5.64 s, *P* < 0.005), whereas the immobility time was significantly longer (260.13 ± 5.64 s, *P* < 0.005) (**Figures 7C, D**). In the FST, the depression group also exhibited reduced struggling time (62.77 ± 4.19 s, *P* < 0.005) and increased immobility time (232.50 ± 4.81 s, *P* < 0.005) (**Figures 7E, F**). Western blot results revealed that compared with control treatment, corticosterone (Cort) treatment significantly downregulated the expression of the synapse-related proteins BDNF and PSD-95 in murine brain tissue (P < 0.005; **Figures 8A–C**).

These findings indicate impaired synaptic function. The expression levels of the mitophagy-related proteins PINK1, Parkin, LC3B-II, and p62 were significantly upregulated in the Cort-treated group (*P* < 0.05 or *P* < 0.005 versus the control group; **Figures 8D-H**). The upregulation of PINK1 and Parkin suggests that corticosterone-induced mitochondrial damage activates the PINK1/Parkin-dependent mitophagy initiation pathway, thereby facilitating the elimination of damaged mitochondria via ubiquitin tagging. Increased levels of LC3B-II, a core marker protein of the autophagosome membrane, indicate increased autophagosome formation. Furthermore, the accumulation of the autophagic substrate p62 suggests an impairment in autophagosome–lysosome fusion and subsequent degradation, thereby impairing autophagic flux. The concurrent shifts in these parameters suggest that the model is characterized by dysregulated mitophagy, marked by excessive initiation coupled with impaired clearance. Consequently, damaged mitochondria are not degraded in a timely manner, potentially exacerbating synaptic injury.

**Figure 9:**
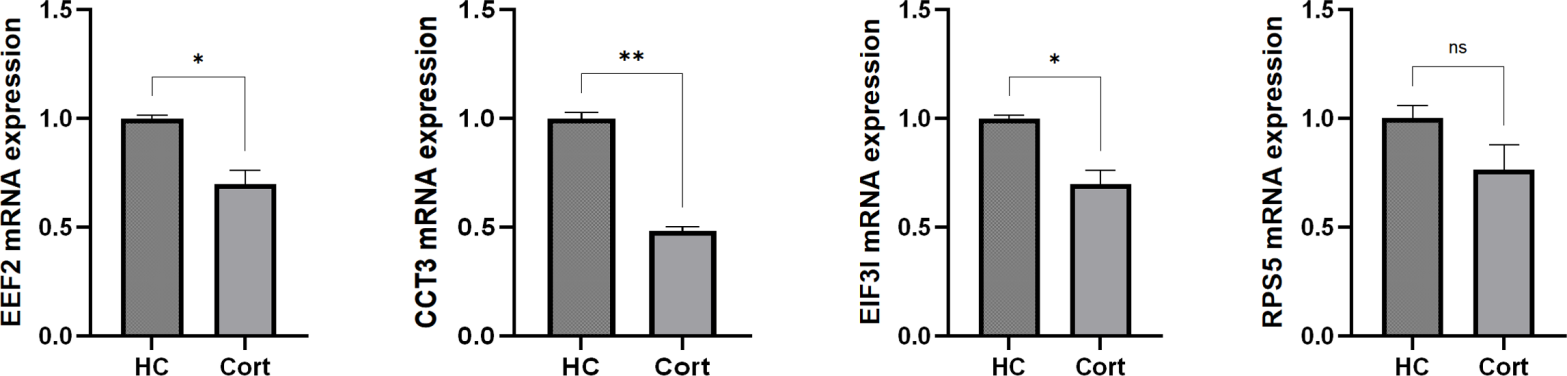
qRT–PCR was used to quantify the relative expression levels of *EEF2*, *CCT3*, and *EIF3I* mRNAs, and the results revealed that these levels were significantly lower than those observed in the control group (ns *P*>0.05, *P < 0.05, **P < 0.005).

Quantitative real−time PCR revealed that corticosterone treatment significantly reduced the mRNA expression of *EEF2* (CT: 19.77 ± 0.04; *P* < 0.05), *CCT3* (CT: 23.86 ± 0.06; *P* < 0.005), and *EIF3I* (CT: 25.29 ± 0.14; *P* < 0.05) in mouse brain tissue. In contrast, the *RPS5* mRNA level (CT: 29.39 ± 0.22) did not significantly differ from that in the control group (*P* > 0.05; **Figure 9**).

## 4 Discussion

This study investigated the core molecular regulatory network underlying the crosstalk between oxidative stress and mitophagy in major depressive disorder (MDD). By integrating an approach involving transcriptome screening based on the training dataset from the GEO database, validation in an independent verification cohort, phenotypic detection in cell models, and multidimensional validation in animal models, we first identified four key genes, namely, *EEF2*, *CCT3*, *EIF3I* and *RPS5*, which are significantly correlated with both oxidative stress and mitophagy. Although *CCT3* and *RPS5* exhibited limited diagnostic performance in the independent clinical validation cohort (AUC ≈ 0.6), which fell short of the threshold required for high-precision diagnostic biomarkers, *EEF2* and *EIF3I* lacked independent clinical diagnostic utility. Nevertheless, using a Cort-induced C8−D1A cell model, we found that the expression levels of these genes correlated negatively with reactive oxygen species (ROS) levels and positively with mitochondrial membrane potential (as assessed by a JC−1 assay), confirming their significant association with elevated oxidative stress and mitochondrial dysfunction. Further validation in animal models revealed that the mRNA expression levels of EEF2, CCT3, and EIF3I were significantly decreased in the Cort-treated group (P<0.05, P<0.005, P<0.05, respectively). Concurrent analysis demonstrated that the expression of the synapse-related proteins PSD-95 and BDNF decreased in the model group, whereas the levels of markers of the mitochondrial mitophagy pathway, including PINK1, Parkin, LC3-II, and the autophagic substrate p62, markedly increased. These findings suggest that abnormal expression of core translational regulatory genes, mediated by alterations in protein synthesis efficiency, may synergistically contribute to the pathology characterized by dysregulated mitochondrial autophagy (excessive initiation and impaired clearance) and reduced synaptic plasticity. On the basis of these results, in this study, a specific molecular regulatory network focusing on translational regulation was developed.

All four core genes identified in this study are involved in protein translation and folding (36–38). These results provide novel molecular evidence supporting the pathological model suggesting a vicious cycle linking energy crisis and proteostasis collapse in MDD. Dysregulated translational control impairs the synthesis of synaptic structural proteins, thereby reducing synaptic plasticity, a key mechanism driving cognitive impairment and emotional dysregulation in MDD (38–40). Specifically, as eukaryotic elongation factor 2, *EEF2* downregulation directly inhibits protein translational elongation (41–43). This finding is consistent with the decreased expression of synaptic markers and neurotrophic factors (PSD-95 and BDNF) observed in this study, suggesting that impaired translational elongation may act as a critical mechanistic link between oxidative stress, dysregulated mitophagy, and synaptic damage.

Consistent with the findings of previous studies, our findings indicate that the diagnostic performance of *CCT3* and *RPS5* was suboptimal in the independent validation cohort (AUC ≈ 0.6). These results align with a well-established principle in biomarker research: the predictive performance of a single gene is often compromised by sample heterogeneity, variability in detection methodologies, demographic variability, and other confounding factors, thereby precluding attainment of the diagnostic threshold for clinical application (44). Although EEF2 and EIF3I did not demonstrate independent diagnostic utility, their significantly altered expression levels in animal models suggest that they may serve as mechanistic therapeutic targets rather than purely diagnostic ones. These findings are consistent with the evolving paradigm in MDD biomarker research, which increasingly prioritizes mechanistic targets over purely diagnostic ones (45, 46).

Oxidative stress is a pathological state caused by an imbalance between intracellular ROS generation and the antioxidant defense system (13, 47). The human brain is particularly vulnerable to oxidative stress owing to its high oxygen consumption, high lipid content, and relative weakness of the antioxidant defense system (48, 49). Molecular evidence has indicated that the expression of the antioxidant enzyme-encoding genes SOD1, SOD2, and GPX1 is significantly downregulated in the brains of patients with MDD (50). Further critical dysregulation is observed in the nuclear factor erythroid 2−related factor 2 (Nrf2) antioxidant pathway. The expression levels of Nrf2 and its downstream antioxidant enzymes (such as heme oxygenase-1 and HO-1) are decreased in the peripheral blood of MDD patients, suggesting that the impaired function of the Nrf2 antioxidant pathway compromises the ability of cells to counteract oxidative stress (16, 51). Nrf2 and mitophagy (PINK1/Parkin axis) pathways exhibit significant reciprocal regulation (52). Decreased Nrf2 activity further exacerbates mitochondrial dysfunction and ROS leakage, thereby establishing a vicious cycle between oxidative stress and mitochondrial damage. Studies have demonstrated that the pathogenesis of major depressive disorder (MDD) involves the interplay among the pathological triangle of neuroimmunity–metabolism–oxidative stress (NIMETOX). This triad establishes a vicious cycle through the “inflammation–oxidative stress loop” and “metabolism–mitochondrial dysfunction axis”, ultimately leading to impaired neural plasticity and impaired emotional regulation (53). Oxidative stress, which serves as a common core mechanism in these conditions, induces reactive oxygen species (ROS) accumulation driven by thyroid hormone deficiency in patients with hypothyroidism and Hashimoto’s disease. Moreover, autoantibodies such as thyroid peroxidase antibodies and anti-α-enolase antibodies in individuals with Hashimoto’s disease can traverse the blood–brain barrier, thereby triggering neuroinflammation and compromising hippocampal synaptic plasticity (54). Patients with coronary heart disease and unstable angina exhibit an integrated phenotypic profile characterized by the metabolism–immunity–oxidation–opioid pathway. Cardiac metabolic abnormalities activate the NLRP3 inflammasome and lead to excessive ROS accumulation, which not only aggravates atherosclerosis but also inhibits hippocampal BDNF expression and subsequently triggers depression (55). Such cross-disease evidence strongly supports the growing consensus that depression is a systemic disease and that oxidative stress serves as a common convergent pathway across multiple diseases. Through bioinformatics analysis, we identified 44 genes associated with oxidative stress in MDD patients who were predominantly enriched in the tricarboxylic acid (TCA) cycle. Kisty reported that elevated ROS levels are not only a consequence of TCA cycle dysfunction but also disrupt cellular energy metabolism by oxidatively modifying cysteine residues of key TCA cycle enzymes and thereby impairing their catalytic activity. It is speculated that DEGs related to oxidative stress may contribute to the pathogenesis and progression of MDD through modulation of the tricarboxylic acid (TCA) cycle pathway (56). We identified 57 DEGs associated with mitophagy, which were significantly enriched in the ribosome pathway, the ubiquitin-mediated proteolysis pathway, and core carbon metabolism modules, which are closely linked to energy reprogramming and translational burden. Enrichment analysis of the mitophagy and oxidative stress pathways revealed that the energy metabolism (carbon metabolism) pathway is involved in both biological processes.

Reported that excessive ROS not only are a consequence of TCA cycle dysfunction but can also disrupt cellular energy metabolism by oxidatively modifying the cysteine residues of key TCA cycle enzymes and impairing their oxidative activity. Further functional and pathway enrichment analyses of these DEGs revealed that oxidative stress-related DEGs are involved in the regulation of carbon metabolism, oxalic acid and dicarboxylic acid metabolism, pyruvate metabolism and the TCA cycle pathway. These findings indicate that oxidative stress-related DEGs may be involved in the occurrence and development of MDD through the regulation of the TCA cycle. Research has indicated that NADPH and α-keto acids detoxify ROS during carbon metabolism (57). Key metabolites involved in carbon metabolism, including acetyl-CoA, pyruvate, and lactate, regulate mitophagy (58). Cellular energy and redox status, as the core output signals of carbon metabolism, serve as the common molecular basis for the regulation of oxidative stress and mitophagy. These findings suggest that carbon metabolism may mediate functional crosstalk between these two pathways and that dysregulation of these pathways may collectively contribute to the pathophysiological alterations associated with MDD. This study confirmed via flow cytometry that the corticosterone (CORT)-induced C8-D1A cell model consistently recapitulates the hallmarks of elevated oxidative stress (increased ROS) and mitochondrial dysfunction (decreased membrane potential) in MDD. This study addresses the lack of phenotypic validation in existing MDD cell models and provides a reliable experimental system for subsequent mechanistic research on MDD at the cellular level.

The core goal of this study is to reveal the correlations between translation regulation-related genes and the interactive network of oxidative stress and mitophagy in MDD, providing a novel molecular perspective for understanding the vicious cycle linking energy crisis and proteostasis collapse in MDD. Downregulated gene expression leads to translational disorders. On the one hand, it leads to reduced synthesis of synaptic proteins, directly compromising synaptic plasticity; on the other hand, it exacerbates mitochondrial dysfunction and ROS accumulation by disrupting the synthesis of mitophagy-related proteins, thus perpetuating a vicious pathological cycle.

Although this study did not identify high-precision diagnostic biomarkers, the screened genes can serve as core components for subsequent combined multigene diagnostic models. The construction of a multiomics diagnostic model through the integration of epigenetic biomarkers and metabolomic data holds promise for improving the accuracy and specificity of MDD diagnosis. Furthermore, as key molecules in the translational regulatory pathway, these genes represent promising targets for the development of novel antidepressants that modulate the translational process. Notably, activating EEF2 kinase or modulating the EIF3 complex can alleviate disruptions in proteostasis and impairments in synaptic plasticity in MDD.

The scope of this study is restricted to correlation analysis. The regulatory roles of the aforementioned genes in oxidative stress, mitophagy, and synaptic function have not been validated via intervention experiments, such as gene knockdown or overexpression; consequently, their causal relationships remain unestablished. Future studies should employ gain- and loss-of-function experiments in cell models to further investigate these functional roles. With the integration of cutting-edge research directions, such as the PINK1/Parkin-dependent mitophagy pathway and the Drp1-mediated endoplasmic reticulum–mitochondria contact (MERCs) regulatory axis, future work must further elucidate the specific regulatory mechanisms of these genes in the pathogenesis of MDD. The sample sizes of the training and validation sets in this study are limited, and MDD patients with different subtypes and disease courses were not represented, which constrains the generalizability of the research findings. Further validation of the expression profiles and diagnostic efficacy of these genes is warranted in larger, independent, multicenter cohorts. Furthermore, a multiomics diagnostic model could be developed by integrating epigenetic biomarkers and metabolomic data to improve the diagnostic accuracy and specificity of MDD. The animal experiments in this study failed to elucidate the direct interaction between the above genes and both the endoplasmic reticulum–mitochondria contact (MERCs) regulatory axis and the PINK1/Parkin pathway. Furthermore, the full regulatory cascade of “molecular–phenotype–behavior” remains incompletely understood. Future research can further investigate the effects of traditional Chinese medicine compounds and small-molecule compounds on alleviating the pathological phenotypes of MDD through the regulation of these genes, thereby promoting the clinical translation of basic research findings.

## Data Availability

The datasets presented in this study can be found in online repositories. The names of the repository/repositories and accession number(s) can be found in the article/**Supplementary Material**.
