# Supplementary material for: Integration of transcriptome profiling to identify key genes involved in the interplay between oxidative stress and mitophagy in major depressive disorder, followed by multidimensional phenotypic validation
Source: Front Psychiatry. 2026 Jun 19;17:1814473. doi: 10.3389/fpsyt.2026.1814473 (PMC13329731; doi:10.3389/fpsyt.2026.1814473)
Supplement: Supplementary file 1 [file Supplementaryfile1.doc]

# =============================================================================

# GSE98793 外部验证脚本

# 数据集: GSE98793 (GPL570, Affymetrix HG-U133 Plus 2.0)

# 样本量: MDD = 128, HC = 64 (共192例)

# 组织: Whole blood

# 候选基因: EEF2, CCT3, EIF3I, RPS5

# 批次校正: ComBat (sva包)

# 多重检验校正: BH法，m = 4（仅对4个候选基因检验）

# 结果: CCT3 (padj=0.027**), RPS5 (padj=0.012**)

# =============================================================================

# ── Step 1: 安装/加载依赖包 ──────────────────────────────────────────────────

if (!requireNamespace("BiocManager", quietly = TRUE))

install.packages("BiocManager")

pkgs_bioc <- c("GEOquery", "limma", "sva", "hgu133plus2.db")

for (p in pkgs_bioc) {

if (!requireNamespace(p, quietly = TRUE)) BiocManager::install(p)

}

pkgs_cran <- c("ggplot2", "ggpubr", "pROC", "cowplot", "dplyr", "stringr")

for (p in pkgs_cran) {

if (!requireNamespace(p, quietly = TRUE)) install.packages(p)

}

suppressPackageStartupMessages({

library(GEOquery); library(limma); library(sva)

library(hgu133plus2.db)

library(ggplot2); library(ggpubr); library(pROC)

library(cowplot); library(dplyr); library(stringr)

})

# ── Step 2: 下载数据 ──────────────────────────────────────────────────────────

cat("Downloading GSE98793...\n")

gse <- getGEO("GSE98793", GSEMatrix = TRUE, getGPL = TRUE,

destdir = ".")[[1]]

pdata <- pData(gse)

cat("Samples:", nrow(pdata), "\n")

# ── Step 3: 提取分组标签 ──────────────────────────────────────────────────────

# characteristics_ch1 包含 "CASE" 或 "CONTROL"

group <- ifelse(grepl("CASE|MDD|case", pdata$characteristics_ch1), "MDD", "HC")

cat("Group counts:\n"); print(table(group))

# ── Step 4: 提取批次信息 ──────────────────────────────────────────────────────

# GSE98793 包含两个子研究 (GSE81152 + GSE92538)，用 series_id 作为批次

batch <- as.factor(pdata$series_id)

cat("Batch counts:\n"); print(table(batch))

# ── Step 5: 表达矩阵预处理 ───────────────────────────────────────────────────

expr <- exprs(gse)

cat("Expression range:", range(expr, na.rm = TRUE), "\n")

# log2 转换（如未转换）

if (max(expr, na.rm = TRUE) > 100) {

expr <- log2(expr + 1)

cat("Log2 transformed.\n")

} else {

cat("Already log-scale.\n")

}

# 分位数归一化

expr <- normalizeBetweenArrays(expr, method = "quantile")

cat("Quantile normalization done.\n")

# ── Step 6: 探针注释 → 基因符号 ──────────────────────────────────────────────

fdata <- fData(gse)

# 方法1: 直接从 fData 提取 Gene Symbol

gene_sym <- str_trim(str_split_fixed(fdata$`Gene Symbol`, "///", 2)[, 1])

gene_sym[gene_sym == ""] <- NA

# 过滤无注释探针和 ncRNA

is_ncrna <- grepl("^MIR|^SNOR|^LINC|^LOC", gene_sym)

keep <- !is.na(gene_sym) & !is_ncrna

cat("Probes kept:", sum(keep), "of", nrow(fdata), "\n")

expr_filt <- expr[keep, ]

sym_filt <- gene_sym[keep]

# 折叠到基因水平（取均值）

expr_gene <- do.call(rbind, lapply(

split(seq_len(nrow(expr_filt)), sym_filt),

function(idx) {

if (length(idx) == 1) expr_filt[idx, ]

else colMeans(expr_filt[idx, ], na.rm = TRUE)

}

))

cat("Genes after collapsing:", nrow(expr_gene), "\n")

# ── Step 7: ComBat 批次校正 ───────────────────────────────────────────────────

cat("\nRunning ComBat batch correction...\n")

mod <- model.matrix(~ as.factor(group)) # 保护分组效应

expr_corrected <- ComBat(

dat = expr_gene,

batch = batch,

mod = mod,

par.prior = TRUE,

prior.plots = FALSE

)

cat("ComBat done.\n")

# ── Step 8: 候选基因统计检验 ─────────────────────────────────────────────────

candidates <- c("EEF2", "CCT3", "EIF3I", "RPS5")

group_bin <- ifelse(group == "MDD", 1, 0)

cat("\n=== 统计检验结果 ===\n")

p_vals <- setNames(numeric(length(candidates)), candidates)

for (g in candidates) {

mdd_v <- expr_corrected[g, group == "MDD"]

hc_v <- expr_corrected[g, group == "HC"]

# Shapiro-Wilk 正态性检验

sw_mdd <- shapiro.test(sample(mdd_v, min(50, length(mdd_v))))$p.value

sw_hc <- shapiro.test(sample(hc_v, min(50, length(hc_v))))$p.value

if (sw_mdd > 0.05 & sw_hc > 0.05) {

test_res <- t.test(mdd_v, hc_v); method <- "t-test"

} else {

test_res <- wilcox.test(mdd_v, hc_v); method <- "Wilcoxon"

}

r <- roc(group_bin, expr_corrected[g, ], direction = ">", quiet = TRUE)

ci_val <- ci.auc(r, conf.level = 0.95)

dir <- ifelse(mean(mdd_v) < mean(hc_v), "DOWN ✓", "UP ✗")

p_vals[g] <- test_res$p.value

cat(sprintf("%-6s | %-8s | HC=%.3f MDD=%.3f | %s | p=%.4f | AUC=%.3f [%.3f-%.3f]\n",

g, method, mean(hc_v), mean(mdd_v), dir,

test_res$p.value, auc(r), ci_val[1], ci_val[3]))

}

# BH 校正：m = 4（仅4个候选基因）

padj_vals <- p.adjust(p_vals, method = "BH")

cat("\n=== BH 校正后 p 值 (m = 4) ===\n")

for (g in candidates) {

sig <- ifelse(padj_vals[g] < 0.05, " **", ifelse(padj_vals[g] < 0.1, " *", ""))

cat(sprintf("%-6s padj = %.4f%s\n", g, padj_vals[g], sig))

}

# ── Step 9: 生成汇总表格 ──────────────────────────────────────────────────────

rows <- lapply(candidates, function(g) {

mdd_v <- expr_corrected[g, group == "MDD"]

hc_v <- expr_corrected[g, group == "HC"]

sw_mdd <- shapiro.test(sample(mdd_v, min(50, length(mdd_v))))$p.value

sw_hc <- shapiro.test(sample(hc_v, min(50, length(hc_v))))$p.value

if (sw_mdd > 0.05 & sw_hc > 0.05) {

test_res <- t.test(mdd_v, hc_v); method <- "t-test"

} else {

test_res <- wilcox.test(mdd_v, hc_v); method <- "Wilcoxon"

}

r <- roc(group_bin, expr_corrected[g, ], direction = ">", quiet = TRUE)

ci_val <- ci.auc(r, conf.level = 0.95)

data.frame(

Gene = g,

Dataset = "GSE98793",

Platform = "GPL570 (Affymetrix HG-U133 Plus 2.0)",

Tissue = "Whole blood",

N_MDD = sum(group == "MDD"),

N_HC = sum(group == "HC"),

HC_mean = round(mean(hc_v), 3),

MDD_mean = round(mean(mdd_v), 3),

Direction = ifelse(mean(mdd_v) < mean(hc_v), "DOWN", "UP"),

Consistent = ifelse(mean(mdd_v) < mean(hc_v), "Yes", "No"),

Test_method = method,

p_raw = signif(test_res$p.value, 4),

padj_BH_m4 = signif(padj_vals[g], 4),

Significant = ifelse(padj_vals[g] < 0.05, "Yes (**)", "No"),

AUC = round(auc(r), 3),

AUC_95CI_lo = round(ci_val[1], 3),

AUC_95CI_hi = round(ci_val[3], 3),

stringsAsFactors = FALSE

)

})

res_df <- do.call(rbind, rows)

write.csv(res_df, "Table_GSE98793_Validation.csv", row.names = FALSE)

cat("\nTable saved: Table_GSE98793_Validation.csv\n")

# ── Step 10: 箱线图 ───────────────────────────────────────────────────────────

padj_label <- function(padj) {

if (padj < 0.001) "padj < 0.001"

else if (padj < 0.05) paste0("padj = ", formatC(padj, digits = 3, format = "f"))

else paste0("padj = ", formatC(padj, digits = 2, format = "f"))

}

expr_df <- data.frame(t(expr_corrected[candidates, ]),

Group = group, check.names = FALSE)

plot_box <- lapply(candidates, function(g) {

df_g <- data.frame(Expr = expr_df[[g]],

Group = factor(group, levels = c("HC", "MDD")))

padj <- padj_vals[g]

lbl <- padj_label(padj)

col <- if (padj < 0.05) "#C0392B" else "grey40"

ymax <- max(df_g$Expr) * 1.07

ggplot(df_g, aes(x = Group, y = Expr, fill = Group)) +

geom_boxplot(width = 0.5, outlier.shape = 21, outlier.size = 1.5,

alpha = 0.85, color = "grey30", linewidth = 0.4) +

geom_jitter(width = 0.14, size = 1.0, alpha = 0.45,

aes(color = Group)) +

scale_fill_manual(values = c("HC" = "#3498DB", "MDD" = "#E74C3C")) +

scale_color_manual(values = c("HC" = "#2980B9", "MDD" = "#C0392B")) +

annotate("text", x = 1.5, y = ymax,

label = lbl, size = 3.6, color = col, fontface = "bold") +

labs(title = bquote(italic(.(g))),

subtitle = "GSE98793 (ComBat-corrected, n = 192)",

x = NULL, y = "Expression (log2)") +

theme_bw(base_size = 12) +

theme(

text = element_text(family = "sans"),

plot.title = element_text(hjust = 0.5, face = "bold.italic", size = 13),

plot.subtitle = element_text(hjust = 0.5, size = 8.5, color = "grey50"),

legend.position = "none",

panel.grid.minor = element_blank()

)

})

p_box <- plot_grid(plotlist = plot_box, nrow = 2, ncol = 2,

labels = LETTERS[1:4], label_size = 13)

ggsave("Fig_GSE98793_Boxplot.png", p_box,

width = 10, height = 9, dpi = 300, bg = "white")

cat("Boxplot saved: Fig_GSE98793_Boxplot.png\n")

# ── Step 11: ROC 曲线 ─────────────────────────────────────────────────────────

colors_roc <- c("EEF2" = "#E74C3C", "CCT3" = "#3498DB",

"EIF3I" = "#27AE60", "RPS5" = "#9B59B6")

png("Fig_GSE98793_ROC.png", width = 2400, height = 2000, res = 300)

par(mfrow = c(2, 2), mar = c(4, 4.2, 3.5, 1.5), family = "sans")

for (g in candidates) {

r <- roc(group_bin, expr_corrected[g, ], direction = ">", quiet = TRUE)

ci_val <- ci.auc(r, conf.level = 0.95)

padj <- padj_vals[g]

sig_str <- if (padj < 0.05) paste0("padj = ", formatC(padj, digits = 3, format = "f"), " **")

else paste0("padj = ", formatC(padj, digits = 2, format = "f"))

plot(r, col = colors_roc[g], lwd = 2.5,

main = paste0(g, " (GSE98793)\nAUC = ", round(auc(r), 3),

" [", round(ci_val[1], 3), "–", round(ci_val[3], 3), "]"),

cex.main = 0.95, font.main = 2,

xlab = "1 – Specificity", ylab = "Sensitivity",

legacy.axes = TRUE)

abline(a = 0, b = 1, lty = 2, col = "grey60")

legend("bottomright",

legend = c(paste0("AUC = ", round(auc(r), 3)), sig_str),

col = c(colors_roc[g], if (padj < 0.05) "#C0392B" else "grey40"),

lwd = c(2, NA), lty = c(1, NA),

bty = "n", cex = 0.85)

}

dev.off()

cat("ROC curves saved: Fig_GSE98793_ROC.png\n")

cat("\n=== 分析完成 ===\n")

cat("输出文件:\n")

cat(" Table_GSE98793_Validation.csv\n")

cat(" Fig_GSE98793_Boxplot.png\n")

cat(" Fig_GSE98793_ROC.png\n")
